# A journey without maps—Understanding the costs of caring for dependent older people in Nigeria, China, Mexico and Peru

**DOI:** 10.1371/journal.pone.0182360

**Published:** 2017-08-07

**Authors:** Rosie Mayston, Peter Lloyd-Sherlock, Sara Gallardo, Hong Wang, Yueqin Huang, Veronica Montes de Oca, Peter Ezeah, Mariella Guerra, Ana Luisa Sosa, Zhaourui Liu, Richard Uwakwe, Maëlenn M. Guerchet, Martin Prince

**Affiliations:** 1 Health Service and Population Research Department, King’s College London (Institute of Psychiatry, Psychology and Neuroscience), London, United Kingdom; 2 School of International Development, University of East Anglia, Norwich, United Kingdom; 3 Instituto de la Memoria y Desordenes Relacionados, La Molina, Lima, Peru; 4 Peking University, Institute of Mental Health, Beijing, China; 5 National Institute of Neurology and Neurosurgery of Mexico, Autonomous National University of Mexico, Delegacion Tlalpan, Mexico City, Mexico; 6 Department of Sociology/Anthropology, Nnamdi Azikiwe University, Awka, Anambra State, Nigeria; Boston University School of Public Health, UNITED STATES

## Abstract

**Purpose of the study:**

Populations in Latin America, Asia and sub-Saharan Africa are rapidly ageing. The extent to which traditional systems of family support and security can manage the care of increased numbers of older people with chronic health problems is unclear. Our aim was to explore the social and economic effects of caring for an older dependent person, including insight into pathways to economic vulnerability.

**Design & methods:**

We carried out a series of household case studies across urban and rural sites in Peru, Mexico, China and Nigeria (n = 24), as part of a cross-sectional study, nested within the 10/66 Dementia Research Group cohort. Case studies consisted of in-depth narrative style interviews (n = 60) with multiple family members, including the older dependent person.

**Results:**

Governments were largely uninvolved in the care and support of older dependent people, leaving families to negotiate a ‘journey without maps’. Women were de facto caregivers but the traditional role of female relative as caregiver was beginning to be contested. Household composition was flexible and responsive to changing needs of multiple generations but family finances were stretched.

**Implications:**

Governments are lagging behind sociodemographic and social change. There is an urgent need for policy frameworks to support and supplement inputs from families. These should include community-based and residential care services, disability benefits and carers allowances. Further enhancement of health insurance schemes and scale-up of social pensions are an important component of bolstering the security of dependent older people and supporting their continued social and economic participation.

## Introduction

The populations of low and middle income countries (LMIC) in Latin America, Asia and sub-Saharan Africa are ageing fast, with some of the poorest countries making this transition most rapidly. As populations age, so the prevalence of chronic disease and disability increases [1]. Other societal changes associated with increased wealth and development, such as increased education and workforce participation, particularly among women; later marriages, reduced fertility, increased divorce and separation and rural to urban migration are likely to complicate how societies manage the care of greatly increased proportions of older people relative to adults of working age [2]. In high income countries (HICs), where large populations of older people have become the norm, family care and support of older people takes place within the context of health and social policies, which, to a varying degree, provide a framework of community and residential care that supports, supplements or substitutes inputs throughout dependency in later life, with the aim of mitigating costs and providing an acceptable level of social and material security. In most LMICs, however, long-term care policy continues to be premised on the questionable assumption that it can be left to informal provision of care and traditional social protection [3]. In the context of fundamental societal and demographic change, it remains to be seen to what extent family systems alone are effective in protecting their older relatives from material poverty and associated social disadvantage and exclusion in older age [4].

It has been suggested that structural socioeconomic changes that have led to altered patterns of co-residency have not destroyed extended kin networks but rather have led to a ‘modified extended family’ paradigm, wherein families adapt to geographical dispersion, retaining many of the functions of the original extended kin model [5]. This adapted model may perhaps continue to sustain the support of older adults, perhaps via what Antonucci (1990) posited as a “support bank” extension of reciprocity, where individuals account for and respond to the level of support they give and receive over the life-course [6]. Evidence suggests that the provision of social pensions has positive effects upon the socioeconomic circumstances of older people and their families: easing older people’s dependence anxiety, contributing to household income and enabling investment in income generating activities that have diffuse benefits for the household; reducing the severity of poverty, supporting children’s education and strengthening of reciprocal ties [7]. Nonetheless, implementation has been slow. There is evidence to suggest that without further transformations of labour markets, social pensions alone may do little to address unequal distributions of pension entitlements and therefore have a minimal overall impact upon poverty [8]. Likewise, despite the roll-out of government health insurance packages, no LMIC has achieved universal coverage [9]. Poorer people may be less likely to enrol and many key expenses, including those associated with chronic illness, are excluded from the schemes, thus limiting their impact upon inequity of access and household finances [10].

Previous work from the 10/66 Dementia Research Group (DRG) baseline and incidence surveys provides evidence to suggest that providing care to dependent older people puts the family system under stress, with largely female carers cutting back on or giving up work to care and experiencing psychological strain [11, 12]. Nested within the 10/66 cohort, in the INDEP study, we used mixed methods to explore in detail the economic and social impacts of old age dependence upon households in Latin America, China and Nigeria [13]. Here we present findings from our qualitative study, carried out among 24 families across urban and rural sites in the four INDEP countries. We used a collective case study approach to address four key questions: a) To what extent was the onset of dependence associated with household impoverishment and economic vulnerability? b) What were the pathways between care dependence and changes to household economic status? c) Which factors made households resilient in the face of increased dependence? d) To what extent did this depend on the external policy environment, including the reach of social protection and health services?

## Design & methods

### Settings

The INvestigating the economic and social effects of DEPendence in older age (INDEP) study is nested within the broader 10/66 DRG cohort [14]. The INDEP study was conducted in 10/66 urban and rural survey catchment areas in four countries: China, Peru, Mexico and Nigeria. 10/66 urban sites are located in capital cities in Peru (Lima Cercado & San Miguel), China (Xicheng) and Mexico (Tlalpan). No urban or rural catchment area can be representative of the aggregate characteristics of the city or country as a whole. Urban catchments were selected to be not atypical of the city population: hence, predominately lower socioeconomic status, or mixed neighbourhoods were selected and middle class or professional enclaves were avoided. Employment in these catchments was mixed, including formal work in services and informal working including trade. Rural sites in all countries were selected to be remote from urban areas, with low population density. In general, the socioeconomic status of rural residents is more homogenous than that of urban residents in 10/66 countries. The 10/66 rural catchments: communities in Canete coastal province, Peru; Daxing, 40kms from Beijing in China; villages in Morelos, a mountainous district 70kms from Mexico City; rural communities in Dunukofia, Anambra State, Nigeria are characterised by higher proportions of people working in the informal economy, carrying out activities such as farming, fishing or trade. For detailed description of the pensions and health insurance context in each setting, and the compositional characteristics of the households and older individuals, see our open access protocol paper [13] and paper describing the economic status of INDEP study households [8]).

### Design

The qualitative study comprised a series of case studies, nested within the quantitative cross-sectional survey. Households were eligible for participation if they had an older resident aged over 65 years with needs for care: ascertained during baseline and incidence 10/66 DRG surveys, with needs for care status and age confirmed during INDEP quantitative interview (for further details of criteria used to determine needs for care, see [13]). Qualitative work was led in-country by a qualitative Principal Investigator (PI) who was an experienced sociologist or anthropologist with an interest in ageing (SG- Peru, VM- Mexico, HW-China, PE- Nigeria). The qualitative component of INDEP, including cross-cultural analyses, was co-ordinated by the UK-based qualitative team (P-LS, RM).

Collective case study methodology has been recommended for research where the key objective is to understand mechanisms, thereby offering a superior approach compared to other qualitative methods in terms of addressing our research questions [15]. We selected the collective case studies method as the most appropriate for our subject matter, with the overall aim of achieving multi-faceted understanding, which may be used to test and develop theory [15]. Case studies are characterised as concurrent consideration of multiple cases, in the real-life setting in which they occur. In the context of our study, “cases” were defined as older people with needs for care [15]. Given that old age dependence is a shared experience located within the family system, the context was defined as the households in which the cases were resident and others who played an important role in their care. Our a priori sample size of 6 households per country was arrived at via a combination of theoretical and practical considerations. We examined sample sizes for previous mixed methods case study designs where it was possible to triangulate data [16]. After piloting the qualitative team discussed how best to balance practical considerations (how to ensure consistently rich and thick data across individual interviews) with theoretical considerations: how many case studies would be sufficient to reach saturation point, regarding the point at which no new themes directly related to our research questions emerged from the data. Given the characteristics of our dataset: a priori questions linked to quantitative findings, interviews covering the same topics with multiple participants, we anticipated that 6 case studies in each country would enable us to reach saturation point in terms of broad themes present across the different cultural settings included in our sample[17].

Our aim was therefore to carry out in-depth interviews (IDIs) with the dependent older person and all the key people involved in their care, including those providing practical care, economic support, or making decisions related to care. Main carers were those identified by the key informant (person identified by interviewer as knowing older person best) as the person with primary responsibility for providing care [13]. Other interviewees were selected for participation on the basis of interviewer judgement regarding level of involvement in care. This judgment was based upon: the narratives of the older person and/or main carer; suggestions from the older person, and/or key informant or main carer.

Pilot in-depth interviews (IDIs) were carried out with members of 1–2 households in each INDEP country (January to May 2013). IDIs were narrative, with the interviewer’s main role being to activate participants’ stories about the onset, course and experience of the older person’s dependence (see S1 File, Topic Guide). The narrative style was selected as being closest to the way in which people “naturally” talk about the health problems of their loved ones, in a way that avoids fragmentation or imposition by the interviewer. Narratives facilitate reflection and interpretation of meaning, encouraging participants to select and order salient features [16]. Interviewers prompted participants using questions from the topic guide, only to stimulate expansion related to our research questions, when this was not covered by the initial narratives. Interviews were carried out in participants’ homes. Length of interviews ranged from 25 minutes to 2 hours and 25 minutes. All interviews were audio-recorded and transcribed in local languages before being translated into English.

The cross-national qualitative team (including UK-based social scientists) met after piloting to reflect on initial findings and to finalise methodology for the main phase of data collection. To clarify the context of the case, at the start of the interview, the main carer was asked to construct a map of the key relationships within the household/close family. Households were purposively sampled for inclusion in the qualitative study from the chronic and incident care households. Given that this was the only post-pilot revision to interview methodology, it was decided that pilot interviews could be included in the main dataset. Selection criteria were determined after analysis and discussion of findings from pilot interviews with: a) the cross-national qualitative team and b) quantitative in-country data collection teams. Our aim was to include households with a diverse range of experiences of managing the care of dependent older people. Reflecting upon pilot data, results from previous 10/66 DRG studies and the broader literature, we identified a list of characteristics that we anticipated may have an impact upon key research questions. Using the quantitative dataset, we selected households for participation in the qualitative study in order to maximise our coverage of these characteristics, for example, ensuring that we included some larger households comprising of extended families, as well as some smaller households where older people were living alone as spousal couples. For a full list of criteria, please see our open access protocol paper [13].

### Ethical considerations

The INDEP study protocol has been approved by King’s College London Research Ethics Committee and relevant local authorities in each study site: Memory, Depression Institute and Risk Diseases (IMEDER) Ethics Committee in Peru; Instituto Nacional de Neurología y Neurocirugía Ethics Committee in Mexico; Medical Ethics Committee of Peking University the Sixth Hospital (Institute of Mental Health) in China; Nnamdi Azikiwe University Teaching Hospital Nnewi Anambra State Ethics Committee in Nigeria. We anticipated that a high proportion of the older people who were potential participants in the study would have dementia. In fact, according to 10/66 diagnostic criteria, up to half of the older people in the incident care households and two thirds of those in the chronic care households were affected by dementia. We used an approach similar to that used previously in 10/66 studies: if the older person lacked capacity to consent, the next of kin was asked to provide signed assent. Participation was subject to the older person not showing signs of distress or dissent when the information sheet was read to them. For each household, the index older person or persons were first approached for consent for an individual and informant interview, and invited to nominate a suitable key informant for the household interview. If they did not consent, the household was excluded.

### Analysis

We used a framework approach [18], as this facilitated within case as well as cross-case analysis. The initial coding matrix was jointly developed at the initial meeting of the qualitative team, using pilot interviews from each country, with reference to literature [7, 11, 12] and our a priori research questions. As part of the process of immersion in the data, RM developed household summaries, detailing key events and turning points in the narratives of dependence. Next, RM applied the initial coding matrix to the final dataset, adding supplementary codes as these emerged from the data and grouped them together in categories. To ensure validity, SG applied this refined matrix to a subset of the data and coding was compared. RM identified key themes. Drafts of the paper were circulated to co-authors (qualitative leads) who were asked to comment on the appropriateness and credibility of findings. We have a comprehensive documented audit trail of materials and analysis processes, much of which is open access and available via the UK Data Service: https://discover.ukdataservice.ac.uk/catalogue/?sn=852071&type=Data%20catalogue

## Results

We carried out 60 IDIs in total, distributed across 24 households. See Table 1. For the distribution of interviews and households across INDEP country sites as well as a description of the relationship of interviewees to the dependent older resident.

**Table 1:** Description of INDEP qualitative participants by country.

*Countries*	Peru	Mexico	China	Nigeria	TOTAL
*Households*					
**Total**	**5**	**6**	**6**	**7**	**24**
*Interviewees*					
Older participants	2	2	3	6	13
Spouses	1	1	3	3	8
Children of older people	5	6	7	6	24
Grand-children	0	1	0	2	3
Other	3	1	3	3	10
**Total**	**11**	**11**	**16**	**20**	**60**

### Trajectories

**Fig 1:**
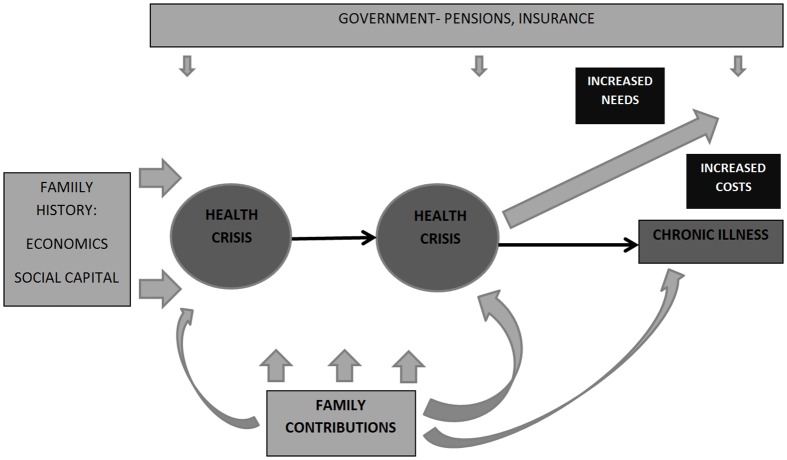
Trajectories and costs of caring for a dependent older person.

Descriptions of trajectories of dependence followed a similar pattern across all sites and were described in terms of crises and periods of chronic poor health (see Fig 1) The overall pattern described was usually one of gradual deterioration in functionality and increasing care needs, interspersed with health crises: characterised as periods of unexpected and significant acute sickness, often requiring hospitalisation and associated with urgent, large and unanticipated out-of-pocket expenditure. Where the older person recovered from crises they often failed to achieve their pre-crisis level of health.

### Who cares and who pays for it?

In most households, women (daughters, daughters-in-law, wives and less commonly grand-daughters and nieces) were the de facto main caregivers. In general, men were identified as household heads and concomitantly as primary decision-makers and economic providers, even in households where women were also working:

*I*: *“Doesn’t it* [mother-in-law’s dependence] *stop your husband from engaging in his own activities even in his business? ”*

*N*: *“He will tell you that there is division of labour and his work is not taking care of women”*

*I*: *“Ok*, *tell us about the division of labour in this household ”*

*N*: *“He said he cannot do any of the things I do”*

*I*: *“Like what?”*

*N*: *“Bathing mama*, *feeding her*, *his major work is contributing money and in decision making*. *If am not around he can only call to ask me where is mama’s drug and he can give her the drugs*.*”*

HH529, Nigeria, daughter-in-law of older lady with dementia

Whilst most caregivers described accepting the care of a dependent older person as part of their role as daughter or wife, a minority of women discussed the dilemmas inherent to their position:

*“Like I say I am working very much*- *I love my job*. *I don´t want to fail my principal*, *my students and their parents*, *I don´t want to stop working*. *I could choose to stop working and stay with her but I resist this*, *maybe like a last choice right? Sometimes that is why she gets depressed”*

HH189, urban Peru, daughter of older lady with stroke

Six households (two in urban Peru, three in Nigeria and one in rural China) had paid for a non-family member to come to the household to take care of the older dependent resident(s). Families explained paying for care, variously as: a response to the onset of care needs for a second older resident; a temporary solution to increased needs for care, deteriorating illness or perceived inability of the main caregiver to cope. A further three households (in rural and urban Mexico and urban Peru) paid female family members for their work. This minimized the burden of practical work on family members who were working but could afford to pay, whilst the compensation offered to the primary caregiver (who was generally otherwise without income) helped to stabilize the caregiving arrangements:

*“When my grandpa passed away*, *well*, *everyone agreed*. *Who? Well*, *we were going to take turns taking care of her*. *And, then, my aunts said they were going to try to give me a little bit of money so that I would come and take care of her”*.

HH121, urban Mexico, grand-daughter of older woman with dementia

### Consequences of dependence

Severe economic hardship, where households struggled to meet the costs of food and healthcare was most common in the Nigerian case studies (described by interviewees in six out of seven households). For example, in household 504, income came from a daughter-in-law selling oranges at market, a daughter selling *agidi* (corn starch product eaten with pepper soup), and small amounts of money from the church but mainly they would “survive from help that comes from concerned people, sometimes we go to borrow”. However, these funds did not necessarily meet household needs:

*“Her sickness affects this family particularly financially*. *Most times, we don’t always have the money for her treatment*. *In the area of feeding her, due to selective feeding pattern as a result of diabetes, we can’t always afford the types of food her ailments require her to eat”*

HH504, Nigeria, daughter of older woman with diabetes and stroke

Seven households (in China, Mexico and Nigeria) described limiting food, other household consumption or access to healthcare for the older person or other family members because of lack of funds. All but one of these households were indebted prior to making the decision to limit healthcare use and expenditure. In the case of the Chinese household, an older couple who were estranged from their two sons and survived on pensions and contributions from their daughter, this restriction was perceived to have had an adverse impact upon the health of the older person:

*“I borrowed money from our daughter*. *I thought I would pay her back after reimbursement*. *That’s why he became disabled -the Doctor said the results of examinations were clear*, *but we could not afford any more* [assessment and treatment] *and then we went home”*

HH385, rural China, wife of older man with stroke

Caregiving responsibilities and filial duty decreased earning potential and curtailed career development. Involvement in caregiving influenced the type of employment undertaken, with caregivers changing jobs to take on work that was part-time, flexible and/or close to home. As a result, caregivers often worked in jobs that were less well paid than previous posts and for which they were over-qualified. This was particularly salient when the older person was living in rural settings:

*“The sickness now controls us*, *including our movement…Like me*, *I was staying in Abuja*, *I came back home instead of staying there and making more money- only to lose my parents to the cold hands of death”*

HH406, Nigeria, son of frail older man

### Balancing the needs of multiple generations: Pathways to economic vulnerability and ways of coping

Across all our sites, there was an expectation from the older and younger generations that children of the older person should meet costs of care. Caring for older relatives, through economic or practical contributions, was perceived as a way of giving back and conveying appreciation for the older generation’s earlier investments:

*“The motive*? *Because my grandmother always looked over us*, *since we were kids and I’m grateful for that and I love her a lot because* [voice begins to crack], *when we didn’t have anything to eat*, *she fed us*, *she took care of us”*

HH121, urban Mexico, grand-daughter of older woman with dementia

However, there was extensive variation within and among sites as to whether, and how, in practice these obligations were met. Large out-of-pocket expenditure associated with health crises often necessitated one-off contributions from multiple sources, which were usually co-ordinated by members of the household where the older person was living at the time of the crisis. This was achieved with varying degrees of success and associated strain. In the context of chronic care needs, the reliability of these regular contributions (such as someone paying utility bills) was appreciated: “basically that’s a huge help to us” (HH86, urban Peru, daughter of an older man with Alzheimer’s Disease whose brothers paid electricity and phone bills). In contrast, the unpredictability of ad-hoc gifts of food, medicine or cash meant that their value was ambiguous: they were felt to contribute little to household security:

*“If they* [his children] *stop helping me*, *they stop helping her*… *she still gets some help*, *yes*, *but sometimes they don’t give us anything*: *it’s been a while*. *When they can*, *they do*, *but it’s very little*…*I’d rather stretch out what they give me*. *If I had ten thousand pesos*, *well*, *yeah*, *that would be great*..*even if they didn’t give me anything*, *I would have somewhere to make money from*. *Sometimes I’m close to running out*, *but I have to figure out how I can make it stretch”*

HH66, rural Mexico, husband of older woman with dementia

Household compositions were fluid and responsive to changes in the status and needs of family members. The older person moving from one household to another often led to changes in contributions, with the responsibility for amassing sufficient funds generally falling upon the primary caregiver in the household where the dependent older person resided. Therefore, decisions made to address needs for care incurred additional costs, without automatically attracting supplementary income to cover these. Sometimes changes to households and contributions were directly related to the status of the older person, but extraneous factors with economic consequences were also important, including divorce (generally of the daughters of the older people) or changes in employment status (usually the sons of the older people). Relocation of younger family members to the older person’s household was often framed as a way of addressing the problems of old and young alike: providing a logical solution to the care needs of the older generation and offering the younger generation a cheaper, more secure place to live. Often, this resulted in the older person financially supporting their descendents. This became problematic in cases where there were many dependents reliant upon a small income, for example, one of our older participants had become economically responsible for his widowed daughter and her children:

*“The death of her husband has compounded my problems*, *I take care of them and her six children*. *They are mostly girls and some of them are serving as housemaids and house-helps for people in various towns in Nigeria”*

HH208, Nigeria, older man with stroke

Sometimes, the older person’s residence changed. In the case of two of our Chinese households, to ensure that the costs and other burdens of care were shared, it had been agreed that the older person would move households every 3 or 12 months, respectively. In one of these households, this arrangement was as a result of an ‘advanced directive’ by the older people themselves. The older lady had specified that if she died first, care of her husband should be shared between their sons through the mechanism of him moving between their households every 3 months: “it’s when my mum was still alive. They decided this together. We respect their opinion and thought it was fine” (HH722, China, Son of older man with stroke).

Children of the dependent older person recognised the security offered by the potential monetisation of assets accumulated by the older generation (including houses, land and savings). Those without assets that could be monetised were largely dependent upon the results of the labour of household members. This left them vulnerable to unexpected events and disruption to work as day-to-day survival was for the most part dependent upon daily earnings of household members:

*“For instance*, *today I didn’t go to break fire wood today because something has bitten my legs and since then I didn’t go to work*. *As such*, *we have not eaten*, *why…*? *Because I have used up all the money I earned*. *We survive through ‘onye lua olie’* [if you work you eat]”

HH204, Nigeria, son of older lady with probable dementia and blindness

In their later years, many older people were left with little choice but to depend largely on the support of their relatives. Although many healthy older people continued to participate in income generating activities, for example, trading produce, their capacity for earning was limited, for example by: reduced energy, lack of mobility and capacity for work, caregiving duties, exclusion from employment, lack of access to credit:

*Interviewer*: *“They* [building contractors] *don’t want* [to employ] *you any more*?*”*

*Juan*: *“No, not anymore, definitely not anymore, not when you’re eighty-two*. *Hmm, well, it’s a risk for them, for the contractors”*

[later in the interview]

*Juan*: *“ …like I said to my daughter-in-law, I tell her, with the few cents I have, I keep making it last and well…*

*Liana* (*Juan’s daughter-in-law*):

“He stretches it out, he stretches it, he stretches his money“

*Juan*: *“When it’s gone and I need to ask for a loan, I will go and I ask for it. Maybe $5,000 pesos or more, but how am I going to pay them back if I don’t work*?*”*

HH66, rural Mexico, husband of older woman with dementia

In some cases, provision of intergenerational support was complicated by long-term discord between family members:

*“I asked them [her sons] to take their father to the hospital*. *It was me who paid for the transportation and meals for them*. *I also paid the registration fee and the expense of the ultrasound*. *My oldest son hopes I won’t contact him anymore if such things happen*. *He also said I would fail* [to obtain money], *even if I managed to contact him”*

HH385, rural China, wife of older man with stroke

Given that many of the children of the oldest generation were themselves reaching older age, it was common for them to be simultaneously caring for their parent(s) whilst experiencing chronic health problems with attendant costs. The middle generation were often supporting their own children (and sometimes grandchildren) through extended periods of education and/or economic inactivity at the same time as caring for older family members. This sometimes created conflicts of interest between the needs for care and support of older people and perceived needs for education and material goods of the younger generation:

*““If you need me to quit*, *I will quit*, *mom*, *and I will stay with you*.*” She said*, *“But*, *the girls* [her two grandchildren, still in education],*” well*, *yeah*, *the girls*, *I had gone years without working and the economy affected us for ten years*! *So*, *this… well*, *there was not a lot of care for my mom”*

HH11, urban Mexico, daughter of older man with kidney failure

### The contribution of pensions and insurance

In China, for one older couple who lived alone (with some financial support from their daughter), the state pension was deemed essential to their survival. Although not sufficient to completely counteract the costs of chronic poor health, the introduction of social pensions and public health insurance was perceived to be transformative:

*“The crisis is much less of a problem because of all aspects of good national policy*. *Although the subsidy from the government is not big money, we are satisfied*. *Anyway, it is better than nothing”*

HH3273, urban China, daughter of older man with diabetes and stroke

Nonetheless, meeting the upfront costs of healthcare often necessitated borrowing money, usually from family members, the majority of which was then reimbursed via health insurance and subsequently paid back. The many expenses not covered by insurance, such as costs of medication, caretakers to carry out basic nursing duties such as feeding and bathing whilst the older person is hospitalised, Chinese traditional medicine, and travel costs, were perceived to be more problematic:

*“The sum spent on treatment for the stroke was lower than the sum spent on diabetes treatment*. *We spent 100 Yuan for each change of dressings*. *And this treatment can’t be covered*. *Changing dressings has continued for about three years*. *Out brothers and sisters share the cost together”*

HH3273, urban China, daughter of older man with diabetes and stroke

Sometimes, with no social security, families had no choice but to pay for healthcare. However, in Mexico, despite the existence of Seguro Popular, participants expressed a preference for private healthcare, because it was perceived to be a way to avoid the delays in obtaining appointments and queues in the public sector. However, in some cases, the costs of treating a severe, chronic illness were unsustainable. For example, since 2012, Miguel needed dialysis:

*“and every session is stressful because my dad says*: *‘No, let’s not go twice a week*, *let’s just go once a week and we can go on Saturday*, *I don’t feel so bad’*. *No, we will see how we do it but we will go to the bank and get a loan with 5% interest and then I will go and ask for more money to pay the original debt and I keep doing this and I’m over $40000 pesos in debt*, *plus the $200000, but they keep lending me money “*

HH11, urban Mexico, daughter of older man with kidney failure

Several interviewees questioned the value of insurance, due to lack of coverage of some essential medications, for example: “Everyone here buys it because we don’t have any of that. It is useless to have insurance”. Interviewees generally perceived pensions to be of minimal benefit. Some were critical of policies. For example, questioning the fact that the pension for those over 70 years old was only available to those without other pensions, as well as querying the rationale for the much higher pensions in Mexico City: “here it’s not like that, they’re screwing us over”.

## Discussion

Despite expanding numbers of dependent older people in low and middle income countries, our quantitative and qualitative results confirm that the state remains uninvolved in the assessment of needs of dependent older people, the provision of care and the support of informal caregivers. Families who participated in our qualitative study negotiated the deterioration of their older relative’s health as a ‘journey without maps’, using a ‘bricolage’ approach to piece solutions together using resources from within the family system in an attempt to meet the needs of multiple generations. Our case studies demonstrate the weaknesses and inequities inherent to reliance upon this approach and provide an indication of the circumstances that push some older people and their households towards poverty as well as illustrating the consequences of this. A key strength of this nested qualitative research is that it enables us to explore possible mechanisms for quantitative findings.

In the absence of effective provision by the state, security in old age is dependent upon the continued sanctity of norms of intergenerational support situated within family systems. Our findings reveal that these norms are being challenged from a number of interconnected directions, resulting in economic vulnerability of older people and the households in which they are resident. Rapid societal shifts that have occurred as a result of macroeconomic changes are the catalysts for these challenges. These changes have occurred rapidly, during the lifetime of the older participants in the 10/66 Dementia Research Group studies. Older and younger participants universally asserted that parents’ early investment in upbringing, including material, time and emotional inputs “naturally” warranted later reciprocal investments from their children [6]. However, in the context of uncertain macroeconomic forces, as has been observed in the US, the middle generation reported struggling to meet their own aspirations for their nuclear family: the education of children, their own employment and career development [19].

In our quantitative study, we found that healthcare spending was higher and catastrophic spending on healthcare was more common in care households [20]. Qualitative narratives demonstrated the consequences of these high costs for some families. When their ‘bricolage’ approach failed, some families described restricting healthcare use and food consumption in order to limit further expenditure. Even when not catastrophic, trying to provide care for older dependent family members was experienced as an additional uncomfortable stretch of limited household resources. The trajectory of the older person’s health and associated costs played a role in determining how difficult it was to manage costs. Consistent with our quantitative finding that associations with adverse economic outcomes were more pronounced in chronic compared to incident care households, costs of chronic disease were perceived to be more debilitating than low probability, high cost crises such as inpatient events [21]. For some participants, it is clear that even relatively small daily costs associated with living with a dependent older person (transportation, special diets, dressings) tipped the balance towards household insecurity, particularly those where there were other disruptions to reciprocal flows between generations, for example: distant or conflicted relationships with children, children who were economically dependent on the older person, or economically unstable. In these cases, in the absence of adequate and equitable social protection, excluded from earning money or access credit, older participants experienced economic as well as social insecurity which they felt powerless to address [22].

In our quantitative study, we found that income from paid work was lower in care households [20]. Findings revealed narratives of reduced income generation activities and restricted careers for men and women involved in the care of older people. Further research is required in order to understand the potential impact of these perceived constraints. Consistent with the results of previous 10/66 surveys, qualitative results demonstrated that women (daughters and daughters-in-law) continued to carry out the vast majority of the work of caring whilst where men contributed it was primarily as decision-makers or as providers of economic support [23]. However, our qualitative findings suggest that the traditional role of female family members as carers, with men as decision-makers is increasingly contested. Participation in the labour market enabled some women to transgress gender norms, legitimising unilateral decisions about care arrangements and enabling some to opt to continue to work rather than automatically taking up a full-time care-giving role. In fact, our study provides evidence to suggest that for those with means, care-giving of dependent older people is becoming increasingly monetised: as demonstrated by the payment of women outside of the family or payment of female family members to carry out care duties. In the context of a societal shift towards materialist values, payment for care makes sense, as it quantifies the value of this work. It should be noted that even when women were successful in negotiating an altered role for themselves, the end result was the transfer of the traditional woman-as-carer role to another woman in a weaker socioeconomic position. Thus, the delineation of caring as “women’s work” and the supervisory and/or role of men in care arrangements held fast, despite greater flexibility of roles for some women [24].

The only government mechanisms perceived to contribute to the costs of caring for older dependent people were health insurance and pensions. Where available, these were appreciated as a small but reliable component of the ‘bricolage’ approach to overall costs. However, in no setting were these provisions perceived to be sufficient to mitigate the economic impact of meeting the needs of a dependent older person. The disparity between pension levels in urban and rural areas was noted and perceived to be unfair. Findings are consistent with the explanation for our quantitative finding that government transfers were lower in care households. We suggested that this association might reflect life-time exposure to economic disadvantage [25], including informal sector employment and access to social pensions only (rather than more generous schemes associated with formal government employment), all of which contributed to poor health in later life. In other words, social pensions cannot mitigate the impact of inequity acquired over the life course [4]. Several households in China and Mexico reported gaps in insurance coverage, with many of the costs of healthcare for chronic conditions excluded. This is consistent with research on the impact of Seguro Popular (universal health insurance in Mexico, introduced in 2003), where participants described the persistence of economic shocks related to health crises and chronic health problems, even in the context of health insurance [26].

The key limitations of our study relate to the case study design and the cross-cultural, multi-site character of the research. Although the consistency of our findings with quantitative results from previous 10/66 studies and INDEP is reassuring, the generalisability of our findings is unclear. Although efforts were made to reach a consensus about our methodology, the approach between country teams is unlikely to be completely consistent. There was good communication between members of the qualitative team, which promoted familiarity with culturally specific themes, and key differences were apparent. Given the lack of qualitative work related to the experiences and impacts of care dependence in older age in LMIC settings and the timeliness of this topic across INDEP countries, the study team felt that it was important to carry out qualitative work in each study site. Although we estimated that our sample size within and across countries would enable us to reach saturation regarding broad themes, it remains likely that our focus upon cross-cultural analysis in four countries may have excluded some of the more subtle culturally mediated differences between sites.

Our results suggest that health and social care systems are lagging behind socodemographic change in LMIC, leaving families to negotiate a ‘journey without maps’, without the structure and support afforded by various health and social policies in HIC. As a result, family resources are stretched, and the material wellbeing, social participation and security of older people are threatened. Any future map will need to be multi-sectoral, integrating health and social care systems whilst acknowledging the continuing role of family in the lives of many older people. The commitment of governments to develop national policies and plans will be essential to facilitate cohesion. Despite the UN calling for the elimination of social and economic inequalities in access to care for older persons in 2002 and the recent adoption by member states of a global strategy on healthy ageing [27], our findings confirm that progress remains extremely limited. Public health systems remain ill-equipped to address the needs of older people: fragmented, with little or no outreach and a focus upon detection and treatment of acute illnesses. Building capacity within primary care to manage chronic conditions and co-morbidities is crucial to improving the health of older people. One example of this is I-COPE: a World Health Organisation-led programme to develop evidence-based guidelines for the prevention and management of dependence by non-specialist healthcare workers, focussing on problems (such as nutrition, mobility, falls, cognition, mood and behaviour, sensory impairment and incontinence), rather than specific conditions [28, 29]. The design and future implementation of I-COPE is a significant potential step towards building the community-based healthcare component of a future map.

Currently, the emerging care industry in low and middle income countries is informal, unregulated, encouraging untrained and inexperienced women to leave families behind in rural areas to undertake poorly paid and unsupervised care-work, most commonly in the homes of older people [11]. The UN global strategy on healthy ageing highlights the importance of the development of long-term care workforce capacity and acknowledges that this work is currently under-valued. The need for resources, information and training for carers is recognised. Likewise, the importance of collaboration between the private sector, non-governmental organisations and civil society, led by national governments is acknowledged, as is the importance of the input of older people in shaping policy developments [27]. Currently, although there are positive examples of advocacy (eg. National Alzheimers Associations) and community-based support, these tend to isolated from governmental systems or accessible only to a minority (eg. Centros de Adultos Mayors in Peru, which are day centres available only to those who are enrolled on Es Salud- an employment-based insurance system, accessible only to retirees from the formal sector). Although traditionally a last resort for older adults with a lack of family support, demand for residential care is beginning to grow, with a proliferation of private facilities in China, for example, in recent years. However, facilities remain unregulated and therefore standards of care are variable [30]. Although the emerging discourse about long-term care is to be welcomed, there is an urgent need for action regarding the development, implementation and scale-up of cohesive frameworks and leadership to support long-term care which will make a difference to the lives of dependent older people and their families.

The final core component of the map is provision of economic security for older people. Actions from policymakers are still required to support economic and social participation and to mitigate the costs of care. Systems of public support need to be more reactive to changes in the older person’s circumstances, including, for example, the provision of disability benefits and carers allowance [3]. Efforts should be made to both increase enrolment to health insurance schemes and expand the range of services included within packages, in order to minimise the need for damaging out-of-pocket expenditure among older people and their families [10]. Given the intergenerational connectedness of family finances, continued scale-up of social protection for older people will help to incentivise home-based care [31] and avert the worst impacts of the costs of chronic care (such as hunger, indebtedness) and will have diffuse benefits for family members, supporting a positive legacy for younger family members and bolstering intergenerational ties [32].

## Supporting information

S1 File Topic guide. (DOCX)

S2 File Quantitative INDEP manuscript. (DOCX)
