# Supplementary material for: A journey without maps—Understanding the costs of caring for dependent older people in Nigeria, China, Mexico and Peru
Source: PLoS One. 2017 Aug 7;12(8):e0182360. doi: 10.1371/journal.pone.0182360 (PMC5546609; doi:10.1371/journal.pone.0182360)
Supplement: S1 File — (DOCX) [file pone.0182360.s001.docx]

**Appendix GUIDANCE FOR INDEP QUALITATIVE INTERVIEWS**

The aim of the guidance provided in the next few pages is to provide a framework for carrying out qualitative interviews that ensures that:

- Interviews are as rich and as detailed as possible
- Interviews contain information on the topics of interest outlined in the INDEP proposal
- Interviews across the different sites are carried out in a similar way so that we are able to make cross-cultural comparisons
- We collect detailed information on all the important people and events that will enable us to answer our research questions

**Topic Guide**

**[NOTE: Wherever possible, you should try to obtain and read through printed copies of all quantitative questionnaires (prevalence, incidence and INDEP) for the household you are interviewing before the qualitative interview. This will provide you with background information on the household and ensure you are well-prepared for the qualitative interview. You may wish to make notes on key variables of interest to INDEP, for example, debt and employment of household members and ask people about these in the questioning phase of the interview- if they are not mentioned by the participant or are contradicted].[Outline script for interviewers]**

Thank you for agreeing to be interviewed.

As you know, we are interested in finding out about the needs of [name of the older person] and how you as a household live together and take care of one another. We are particularly interested in how your family cope financially.

In order to find out about this, I would like to do three things during the course of the interview. I will ask you about the relationships of important people in your family, I will also ask you to tell me about [name of older person] and how things have been for you and the rest of the family. Finally, I will ask you a few questions about what you have told me. At any time, please feel free to include anything that you feel is important.

Does that sound OK?

**[1. MAPPING RELATIONSHIPS- USE FIGURE 1]**

So, firstly, so that I can get an idea of the important people related to this family:

1. Could you tell me about who lives in your home (names, sex, relationship to you, birth year, employment, location)
2. Can you tell me about your parents, children and partner [if not living in the household]
3. Are there any people who have lived with you in the past 10 years who have since moved out or passed away?
4. Are there any people who you haven’t mentioned and who do not live with you who are an important part of your life, or that of [name of older person]

**[2. NARRATIVE- see notes below]**

So tell me about how things have been. When did you first start to notice changes in [older person’s name] health?

[PROMPTS: and what happened after that/what happened next? And how have things been over the last year/months/weeks. How did that affect you/other people in the house?]

[Interviewer should make notes on Figure 2 about key events- changes in older person’s health, changes in household circumstances and change in circumstance for interviewee]

**[3. QUESTIONS- USE FIGURE 2]**

[If not covered in main narrative] How did [event] affect your finances? How did [event] effect the family’s finances?

[If not covered in main narrative] When [change re. care given to older person] happened, who decided that [change re. care given to older person] should happen? Did everyone agree that this was the best thing to happen?

[If not covered in main narrative] What were the reasons for [change re. care given to older person]? Were there financial considerations related to [change re. care given to older person]?

Apart from what you have told me, is there anything else that has happened in the last 10 years that has had a significant impact upon the finances of the family? [Give examples if needed- someone being ill and therefore not being able to work, someone moving away from the house] [If yes] Could you tell me about that?

NOTES FOR INTERVIEWER

Text in [ ] is meant as guidance for the interviewer. All other text is meant as an outline script (ie. to be said) to the interviewee.

2. NARRATIVE

As much as possible, you should avoid interrupting the participant’s story. However, if they come to a pause, it may be helpful to prompt them to tell you “what happened next”.

3. QUESTIONS

You should refer to notes made on Figure 2 and prompt the participant for more information about important events using the suggested questions, if sufficient detail was not provided in the main narrative.

In particular, you may need to ask specific questions about decision making and financial impacts (key topics for INDEP) in relation to all the important events discussed by the participant- if this is not covered in the main narrative.

**Figure 1. MAPPING RELATIONSHIPS KEY: M=male F=female; NR=non-resident; D=died (include date)**

NOTES ON **GRANDPARENTS/STEP-PARENTS**

**FATHER**

**MOTHER**

NOTES ON **PREVIOUS PARTNERS**

**CURRENT PARTNER**

**INTERVIEWEE**

NOTES ON **CHILD 1’s CHILDREN**

**CHILD 1’s CURRENT PARTNER**

**CHILD 1**

NOTES ON **CHILD 2’s CHILDREN**

**CHILD 2**

**CHILD 2’s CURRENT PARTNER**

NOTES ON **CHILD 3’s CHILDREN**

**CHILD 3**

**CHILD 3’s CURRENT PARTNER**

NOTES ON **CHILD 4’s CHILDREN**

**CHILD 4**

**CHILD 4’s CURRENT PARTNER**

**NOTES ON OTHER IMPORTANT PEOPLE**

**Figure 2. TIMELINE OF KEY EVENTS**

| Year | Change in older person’s health and functional status | Change in household circumstances | Change in circumstances of main carer |
| --- | --- | --- | --- |
|  |  |  |  |
|  |  |  |  |
|  |  |  |  |
|  |  |  |  |
|  |  |  |  |
|  |  |  |  |
|  |  |  |  |
