# Supplementary material for: A journey without maps—Understanding the costs of caring for dependent older people in Nigeria, China, Mexico and Peru
Source: PLoS One. 2017 Aug 7;12(8):e0182360. doi: 10.1371/journal.pone.0182360 (PMC5546609; doi:10.1371/journal.pone.0182360)
Supplement: S2 File — (DOCX) [file pone.0182360.s002.docx]

**A cohort study of the effects of older adult care dependence upon household economic functioning, in Peru, Mexico and China**

**Maelenn Guerchet.** Health Service and Population Research Department, King’s College London. Address: Institute of Psychiatry, Psychology and Neuroscience, P060, De Crespigny Park, London SE5 8AF, United Kingdom. Email maelenn.guerchet@kcl.ac.uk

**Mariella Guerra** Psychogeriatric Unit, National Institute of Mental Health “Honorio Delgado Hideyo Noguchi”, Lima, Peru. Address: Jr. Eloy Espinoza 709, Urb. Palao, San Martin de Porres, Lima, Perú. Email: mariella.guerra.1066@googlemail.com

**Yueqin Huang**. Peking University, Institute of Mental Health, Beijing, China. Address No.51 Hua Yuan Bei Road, Haidian District, Beijing, 100191, P. R. China. Email dengy@mail.tsinghua.edu.cn

**Peter Lloyd-Sherlock**. School of Development Studies, University of East Anglia. Address: School of Development Studies, University of East Anglia Norwich, NR4 7TJ, United Kingdom. Email: p.lloyd-sherlock@uea.ac.uk

**Ana Luisa Sosa**. National Institute of Neurology and Neurosurgery of Mexico, Universidad Nacional Autónoma de México, Mexico City, Mexico. Address: Insurgentes Sur 3877, 14269 Mexico City, Mexico. Email: drasosa@hotmail.com

**Richard Uwakwe** Nnamdi Azikiwe University Teaching Hospital, Nnewi, Anambra State, Nigeria. Email: ruwakwe2001@yahoo.com

**Isaac Acosta**. National Institute of Neurology and Neurosurgery of Mexico, Universidad Nacional Autónoma de México, Mexico City, Mexico. Address: Insurgentes Sur 3877, 14269 Mexico City, Mexico. Email: ixhuetzca@gmail.com

**Peter Ezeah**. Department of Sociology/Anthropology, Nnamdi Azikiwe University, Awka Nigeria. Address: Nnamdi Azikwe University Awka, Anambra State, PMB 5025 , Awka – Nigeria. Email: pezeah@yahoo.co.uk

**Sara Gallardo** Instituto de la Memoria, Depresión y Enfermedades de Riesgo (IMEDER), Lima, Peru. Address: Av. Constructores 1230, La Molina, Lima, Perú. Email saragg84@gmail.com

**Zhaorui Liu**. Peking University, Institute of Mental Health, Beijing, China. Address No.51 Hua Yuan Bei Road, Haidian District, Beijing, 100191, P. R. China. Email: zhaoruiliu@gmail.com

**Rosie Mayston.** Health Service and Population Research Department, King’s College London. Address: Institute of Psychiatry, Psychology and Neuroscience, P060, De Crespigny Park, London SE5 8AF, United Kingdom. Email rosie.mayston@kcl.ac.uk

**Veronica Montes de Oca**. Instituto de Investigaciones Sociales, Universidad Nacional Autónoma de México, Mexico City, Mexico. Email: vmoiis@gmail.com

**Hong Wang**. Peking University, Institute of Mental Health, Beijing, China. Address No.51 Hua Yuan Bei Road, Haidian District, Beijing, 100191, P. R. China. Email: [hwang2010@aliyun.com](mailto:hwang2010@aliyun.com)

**Martin J. Prince***, Health Service and Population Research Department, King’s College London. Address: Institute of Psychiatry, Psychology and Neuroscience, P060, De Crespigny Park, London SE5 8AF, United Kingdom. Email martin.prince@kcl.ac.uk

* Corresponding author

**Purpose of the study:** To study the effect of care dependence among older adult residents on the economic functioning of their households, in catchment area survey sites in Peru, Mexico and China.

**Design and Methods:** Cohorts of households were classified from the evolution of the needs for care of older residents, over two previous community surveys, as ‘incident care’, ‘chronic care’ or ‘no care’, and followed up three years later to ascertain economic outcomes (household income, consumption, economic strain, satisfaction with economic circumstances, healthcare expenditure and residents giving up work or education to care).

**Results:** Household income did not differ between household groups. However, income from paid work and government transfers were lower in care households. Consumption was 12% lower in chronic care households. Healthcare expenditure was higher, and catastrophic healthcare spending more common in care households.

**Implications**: Older people have not featured prominently in global health and development agendas. This study indicates that older people’s needs for care have a discernable impact on household economics, in what are, typically, multigenerational family units. Population ageing will rapidly increase the number of households where older people live, and their societal significance. Building sustainable long-term care systems for the future will require some combination of improved income security in old age; incentivisation of informal care through compensation for direct and opportunity costs; and development of community care services to support, and, where necessary, supplement or substitute the central role of informal caregivers.

The inverse correlation between disability and economic status is well established. In the 49 country World Health Survey (over 200,000 adults aged 18 years and older) disability was more prevalent in the poorest than in the richest wealth quintiles in all countries, with a statistically significant gradient in all but six countries (Hosseinpoor et al., 2013). While disability was more prevalent in lower income countries, the inequality gradient was steeper in high- and upper middle-income countries. However, there has been relatively little research on the links between health, disability and poverty in low and middle income countries (LMIC), particularly the impact of care dependence among older adults. In 2011 a critical review included only 27 relevant publications, just 14 focusing on associations between disability and poverty, four among older adults (Groce et al., 2011). We updated this review from 2011 to present, not limited to LMIC, but restricted to studies focusing on older adults, using the search terms (poverty AND (health OR disability OR dependen*) AND (old* or age*)). Cross-sectional surveys from Latin America (Guerra, Alvarado, & Zunzunegui, 2008; Zunzunegui, Alvarado, Beland, & Vissandjee, 2009; Acosta, Rottbeck, Rodriguez, Ferri, & Prince, 2008) and Asia (Zimmer, 2008; Teerawichitchainan & Knodel, 2015) demonstrate that older people with disabilities are more commonly to be found living under adverse socioeconomic conditions, usually quantified in terms of current household assets. These associations were not confirmed in two studies from Nigeria (Gureje, Ogunniyi, Kola, & Afolabi, 2006; Uwakwe et al., 2009). While direction of causality cannot be determined from cross-sectional studies it is clear that the focus of interest for much of this research was whether adverse economic conditions lead to poor health and disability. Evidence from HIC suggests that unhealthy ageing trajectories may be determined, partly, by early life socioeconomic disadvantage, or its cumulative effects across the life course (Tampubolon, 2015; Agahi, Shaw, & Fors, 2014). Tentative evidence based upon retrospective recall of early life exposures supports similar conclusions from studies in Latin America (Guerra et al., 2008; Zunzunegui et al., 2009).

It is equally possible that the onset of chronic ill health, disability and needs for care in an older person impoverishes their household. Plausible mechanisms include work incapacity, family carers cutting back on paid work, increased costs of living, and the costs of health and formal paid care. Several strands of evidence from HIC support this conclusion. In the USA, pre-retirement disability shocks among those aged 51-56 were associated with declining incomes and increased poverty rates over the subsequent eight years (Dushi & Rupp, 2013). In the national Australian Survey of Disability, Ageing and Carers, those aged 45-64 who had retired early due to ill health were twice as likely to be in income poverty than those retiring for other reasons, with increased risks of income poverty extending to other family members (Schofield et al., 2013). Economists have indirectly modelled the costs of disability among older residents, at household level. In Ireland, these were estimated by comparing the standard of living of households with and without older members living with disability, at a given income, controlling for other covariates (Cullinan, Gannon, & O'Shea, 2013). The additional economic cost amounted to one-third of household income, varying by disability severity, and was proportionately greater in smaller households. These estimates capture neither the direct costs associated with disability, such as health and social care, nor the opportunity costs, such as potential foregone earnings.

Our own 10/66 Dementia Research Group (10/66 DRG) population-based surveys in urban and rural catchment area sites in Latin America, India and China showed a consistent tendency for dependence (needs for care) to be inversely associated with educational level (Sousa et al., 2010). Dementia was the leading contributor to disability and needs for care among older people (Sousa et al., 2009; Sousa et al., 2010). Among carers of older people with dementia, cutting back or giving up work to care was common (Prince, 2004), and strongly associated with role strain (Prince et al., 2012). More detailed studies of the correlates of care dependence in the Dominican Republic and rural Nigeria further attested to the risk of economic vulnerability (Acosta et al., 2008; Uwakwe et al., 2009). The 10/66 DRG mixed methods INDEP study is designed to provide a more detailed picture of social and economic consequences of chronic and incident needs for care in older age in selected LMIC (Mayston et al., 2014). There are three key elements of the quantitative part of the study, conducted in rural and urban sites in Peru, Mexico and China. First, we study social and economic impact at the household level, classifying households according to the needs for care of older residents at the time of the baseline and incidence wave surveys, and introducing a longitudinal perspective by following up the selected households three years after the incidence wave. Finally, we assess economic impact more directly than in previous studies, through household consumption as well as income and assets, indicators of economic strain, and the direct costs of health and social care. At the incidence wave survey, assets were similarly distributed between households subsequently selected for ‘care’ and ‘no care’ groups (Mayston et al., 2014). The accompanying qualitative case studies enable us to explore mechanisms underlying any observed associations between care dependence and household impoverishment, including factors that support economic resilience ([Mayston R](#_ENREF_1)).

**DESIGN AND METHODS**

*Design*

A household cohort study, nested within the prevalence (baseline) and incidence waves of the 10/66 DRG surveys in Peru, Mexico and China. Households were selected on the basis of the needs for care of older residents recorded at baseline and incidence waves (with an interval of 3.5 to 5 years), and then followed up three years after the incidence wave interviews.

*Settings and Participants*

The INDEP study is conducted in 10/66 survey catchment areas in four countries; China, Peru, Mexico and Nigeria (Mayston et al., 2014). The INDEP quantitative cohort study was completed in the urban and rural sites in Peru, Mexico and China (see Annex for further details). The catchment area sites are not nationally representative, nor necessarily representative of the city or rural region where they are located. Urban areas were selected to be predominately lower socioeconomic status, or mixed neighborhoods, avoiding middle class or professional enclaves (Prince et al., 2007). Rural areas were selected to be distant from conurbations, and to include a high proportion of inhabitants with agrarian occupations.

*Sampling*

For the INDEP study, we sampled in each site from among those households where one or more older participants (referred to as ‘index older people’ or IOP) had been interviewed at the baseline and incidence waves, categorizing these households as follows.

1) Incident care households (where all IOP were independent at baseline, but in which one or more had become care dependent by the incidence survey.

2) Chronic care households (with one or more care dependent IOP at baseline, who remained care dependent in the incidence survey).

3) No care households (where all IOP were independent at baseline, and remained so at the incidence survey).

All households meeting criteria for incident or chronic care were selected for the INDEP study. In each site, no care households equivalent in number to the sum of incident and chronic care households were selected at random from all those eligible, batch matched to care households for the age of the oldest resident.

*Household tracing and redesignation*

Tracing for the INDEP study was carried out in 2012, three years after the mid-point of the incidence wave surveys. We envisaged that there would have been changes in household composition and needs for care (Mayston et al., 2014). When all IOP who needed care (in incident or chronic care households) had died, the household was redesignated as a ‘care exit’ household, and only the household interview was completed. When all IOP in no care households had died, the household was excluded from the INDEP study. If all surviving IOP had moved together to another household then this was designated as the household of interest. If two or more IOP had moved to separate households, for care households we followed the IOP with the highest level of needs for care at the incidence wave survey, and for no care households we followed the youngest. Needs for care of all IOPs were reappraised in the INDEP study informant interview. Where needs for care had developed for one or more IOP in no care households, such households were still classified as no care households in the main analysis, but were excluded for the sensitivity analysis (see below).

*Data collection*

For each selected household, we aimed to conduct a household interview with a suitable key informant (usually the head of household), brief interviews with each of the surviving IOP, and an interview with an informant for each IOP for an independent perspective on their health and needs for care. All interviews were conducted masked to household group status.

*Measures*

A full account of the interviews administered in the INDEP study is provided in our open access protocol paper (Mayston et al., 2014), and in the Appendix. Here we summarise measures used for the current analyses. The detailed household interview comprises:

1. Household composition and roles - the age, sex, marital, educational and occupational status of all residents.

2. Economic evaluation

a. A household assets index covering household goods and amenities.

b. Monthly household income, estimated by enquiring about 20 different sources of income and allocating each to an individual resident, or to the household if not specifiable. Income sources were clustered into five groups; pensions, paid work, income from assets, government transfers and private transfers. We then summed after tax income across all sources and all residents, equivalised by dividing by the modified Organisation for Economic Cooperation and Development (OECD) equivalence scale, and converted into 2011 international dollars using PPP exchange rates (The World Bank, 2014).

d. Consumption, 25 items eliciting food consumption (the value or cost of all food consumed at home and outside of the home), household expenses and other personal expenditure, also divided by the OECD equivalence scale.

e. Consistent with convention, health and social care expenses were not included in general consumption, but considered separately. Catastrophic healthcare costs were defined as spending more than10% of household income in the last three months on health care.

f. Indicators of household financial strain over the last three years. The number of indicators endorsed was grouped into three categories for the analyses; none, one and two or more.

g. Subjective assessment of overall financial status; How would you rate the financial situation of this household at present? For the purpose of analysis this was grouped into three categories, very good or good, average, and bad or very bad.

*Analyses*

1. Descriptive analyses, by site, weighted to take account of sampling fractions of care and no care households, and non-response at household level, aiming for generalizability to the incidence phase of the 10/66 surveys in each catchment area site (Mayston et al., 2014; Prince et al., 2007). We summarize, for each site the distribution of household size, composition, and socioeconomic status (household assets, and occupational status of the IOP), as assessed at the time of household selection, and in the INDEP study, and their crude association with household care status

2. Testing the main hypotheses

The general approach for the main analyses was to compare, as exposures, no care households with each of the other three care categories (incident care, chronic care and care exit households). We also compared ‘current care’ households (incident and chronic care households combined) with no care households, omitting care exit households. The preselected outcomes were total household equivalised income, total household equivalised consumption, economic strain, satisfaction with economic circumstances, healthcare expenditure, catastrophic healthcare expenditure, and co-residents giving up work or education to provide care for an older adult. Secondary analyses looked at sub-categories of income (from paid work, and from private and government transfers) and consumption (food consumption). Regression models were selected depending on the distributional characteristics of outcome data (see Annex for further details). All models were adjusted for the potential confounding effects of household composition and economic status (household assets, and occupational status of the IOP) at the time of the incidence wave. The effects of household care status on income from paid work (non-equivalised) were further controlled for the number of adult residents, and on healthcare expenditure and catastrophic healthcare expenditure for the number of child and number of adult residents.

3. Sensitivity analysis. We re-estimated the effects of household care status on the main outcomes, excluding households subject to household changes, and no care households where IOPs had developed needs for care.

RESULTS

One thousand three hundred and fifty-four households were selected for the INDEP nested cohort study, on the basis of needs for care for older adults observed in the baseline and incidence wave 10/66 surveys (Table 1). Of these, 493 were incident care households, and 189 chronic care households; 672 age-matched no care households were selected, slightly fewer than the planned one no care household per care household since insufficient age-matched no care households were available in urban Mexico and urban China. Consistent with the study protocol, we then reclassified the households based upon their composition when revisited for the INDEP survey. Sixty-eight (10%) of the no care households were redesignated as ‘lost’ since all older residents had died, and these were excluded from the INDEP survey. 199 (40%) of the incident care households and 89 (47%) of the chronic care households were redesignated as care exit households, since all of the older persons requiring care had died. Therefore, the final household classification for the main analysis comprised 1286 eligible households; 604 control households, 294 incident care households, 100 chronic care households and 288 care exit households.

Household interviews were completed for 872 of the 1286 eligible households (68%), with response rates varying from 52% (China urban) to 89% (China rural) (Table 2). Household interviews were completed on 424 no care households (70% of those eligible), 227 incident care households (77%), 67 chronic care households (67%) and 154 care exit households (54%). Where household interviews were completed, we were generally also successful in interviewing surviving older residents. All those eligible were interviewed in 93% of households with surviving older residents, and at least some eligible older residents were interviewed in a further 6% of such households.

The weighted sociodemographic characteristics of the 872 households with completed household interviews are summarized in Table 3. In all sites the mode was for older people to live in multigenerational households with younger adults, and, often, children under the age of 16. The urban China site stood out as having smaller households, a higher proportion of households where older people lived without younger adults (39.2%), and a very low proportion of households with co-resident children (8.3%). The baseline socioeconomic status of households tended to be higher in urban than rural sites, although China rural households were relatively asset rich despite low levels of occupational attainment. Of these characteristics, only household living arrangements were associated with household care status; specifically, older people living alone (9.4% of all households) were under-represented in incident and chronic care groups. Household changes occurred when index older people were followed from the household originally selected to another location to which they had moved since the 10/66 survey. This affected 89 households (10.2%). Care households were marginally more likely to have been subject to household changes, but this was a non-significant trend. Overall, there was very little change in household size from baseline. Neither change in household size, nor assets at follow-up differed by original household care status.

*Tests of the main hypotheses*

While there was no evidence that total household income differed between care and no care household groups, there was a trend for income from paid work to be lower in incident and chronic care households, with a statistically significant pooled effect for the combined current care group (CR 0.88, 95% CI 0.78-1.00, I^2^=44.5%) (Table 4). Income from external sources (private transfers) was similar between all groups, with a trend towards lower levels in care exit households (CR 0.80, 95% CI 0.61-1.05, I^2^=0.0%). Income from government transfers was lower for both incident and chronic care households compared with no care households.

Total household expenditure was lower in chronic care households compared with no care households (CR 0.88, 95% CI 0.77-0.99, I^2^=0.0%), but not in incident care households. A similar trend was apparent for food consumption. There was a trend towards more indicators of economic strain among care households (CR 1.37, 95% CI 0.97-1.92, I^2^=53.3%). Dissatisfaction with economic circumstances was more prevalent in chronic care than in no care households (CR 1.74, 95% CI 1.02-2.97, I^2^=66.8%).

Healthcare costs were significantly higher among care households in some sites (particularly the urban sites in Peru and China), and in the pooled estimate (CR 1.55, 95% CI 1.26-1.90, I^2^=73.4%) but with considerable heterogeneity among sites (Table 5). Catastrophic healthcare spending was significantly more likely in care households (PR 1.64, 95% CI 1.20-2.22, I^2^=16.3%), with much less heterogeneity of effect between sites. The striking finding in this section of the analysis was that while elevated household healthcare spending was apparent across incident and chronic care households, household healthcare costs in care exit households were significantly lower than in no care, suggesting an important effect of the presence of older household members, in addition to any effects of needs for care. Compared with no care households, not working, or giving up education to care for an older household member was considerably more common in both incident care (PR 2.08, 95% CI 1.37-3.16, I^2^=32.3%) and chronic care households (PR 2.22, 95% CI 1.43-3.43, I^2^=48.3%).

In the sensitivity analysis, we limited the analysis to those households that were stable from the incidence wave (hence excluding the effect of household changes), and also excluded no care households where older residents were found to have developed needs for care (online Table 1). Patterns of association, and effect sizes were generally similar to those from the main analyses. However, when comparing care households with no care households, the effects of care dependence on economic strain (OR 1.64, 95% CI 1.11-2.44, I^2^=60.6%) and dissatisfaction with economic circumstances (OR 1.50, 95% CI 1.04-2.18, I^2^=60.6%) were clarified.

DISCUSSION

In our cohort study in rural and urban catchment area sites in Peru, Mexico and China, we classified households according to the needs for care of older residents across two waves of population surveys. At the second of these waves there were no differences in household assets between households defined as providing ‘no care’, ‘incident care’ or ‘chronic care’. However, when followed up three years later for this nested INDEP cohort study we found that while total household incomes were similar between groups, income from paid work and government transfers was lower in care than in no care households, expenditure on healthcare was higher, and catastrophic healthcare expenditure more common. Consumption was lower in chronic care than no care households, but similar in incident care households.

The impoverishing effects of older adult care dependence are widely discussed, but little studied in LMIC. In these settings, social and economic protection for older people is much more limited than in HIC, with low pension coverage, and a high reliance on out-of-pocket payments for healthcare. There are, furthermore, no structured systems of social care to support, supplement or substitute for family informal care if they lack capacity to meet the demand. New government policies (for example introduction of social pensions, conditional cash transfers, and health insurance), and economic development have led to some recent improvements for older people in the INDEP study countries, but gross inequalities persist determined mainly by the limited reach of the formal labour market and the access it brings to government contributory pension and health insurance schemes (Prince et al., 2016).

The main strength of our study is its longitudinal perspective. Since household exposure to older adult care dependence was established over a three to five year period, ending three years before the assessment of economic outcomes in the nested INDEP study, it is more likely that economic disadvantage has resulted from care needs, rather than vice versa. In further support of this direction of causality, household assets (a long-term indicator of household economic status) were similar among household categories, and associations with lower consumption, economic strain and dissatisfaction with economic circumstances were all more prominent for the chronic care than the more recently incident care group, suggesting a cumulative effect of care dependence over time. Increased direct costs of health and social care for the care dependence households provide one likely explanation for the lower consumption in chronic care households despite similar total household incomes. The plausibility of this explanation is strengthened by its consistency with mechanisms suggested by the narratives of participants in our qualitative study ([Mayston R](#_ENREF_1)). Importantly health and social care costs were assessed at household level, and not only for the IOP. Excessive care demands are known to have an adverse effect on caregiver health (Fonareva & Oken, 2014). The rich description of the health circumstances of the IOP from the previous two waves of 10/66 surveys validated the classification of no care, incident care and chronic care households, with a high prevalence of dementia and stroke among IOP in chronic care households at both time points, and in incident care households at the incidence wave, and a rising mean disability score in incident care households (Mayston et al., 2014). A the incidence wave survey, dementia affected up to half of IOP in the incident care households, and two-thirds in the chronic care households, underlining the typically chronic and progressive nature of needs for care. Health conditions and disability give rise to the needs for care that may lead to economic adversity, and are therefore not controlled for in the analyses presented here. However, we did use previous survey data to control for longer-term household economic status and composition.

The main limitations of the study are the catchment area sampling, the changes of residence and health status, and attrition. The catchment area sampling limits generalisability, since the catchment areas, although carefully characterised, may not be representative of urban or rural settings in general in the countries concerned. Attrition arose for several reasons. First, deaths of care dependent older people lead to redesignation of incident and chronic care households as ‘care exit’. This substantially reduced the numbers of incident and, particularly, chronic care households, but did allow us to assess the economic status of such households after care demands had ceased. A smaller number of no care households were lost because of the deaths of all IOP. Second, only 68% of eligible households could be traced and interviewed, much of the non-response occurring in the ‘care exit’ households. Other than in this group, refusal was rare, and most non-response arose from difficulties in tracing the relevant IOP to the household where they now resided, particularly in the urban China catchment area, due to extensive redevelopment around the time of the Beijing Olympics. The extent and direction of any resulting bias is difficult to determine. The reallocation of care households to the care exit group, coupled with losses to follow-up reduced the power and precision of the analyses that we conducted, as indicated by the breadth of the 95% confidence intervals. This meant that some of the analyses were relatively underpowered, particularly at site level. Sample size and power were limited by the availability of care households at the incidence wave, all of which were selected for the INDEP study. Overall 8.9% of households were subject to household change, due to relocation of IOP. However, this applied to 35.1% of households in rural China, and 13.7% in rural Mexico, and was relatively uncommon in other sites. For the most part the household changes resulted from the IOP moving into another household, rather than the whole household moving en masse to another location. This was a common theme in the qualitative research, particularly for rural China, where older people with needs for care sometimes rotated among their children’s homes to share care demands and costs, or moved to another household in the extended family network where those demands could be more conveniently and adequately met ([Mayston R](#_ENREF_1)). It is likely that these household changes, and changes in household composition when incoming residents bring additional income or capacity to care, are informal mechanisms that mitigate the economic and social impact of older adult care dependence. This issue seems not to have been considered in previous research, probably because of the preponderance of cross-sectional studies. We conducted a sensitivity analysis, in which, following the exclusion of households subject to household changes, most of the associations with economic disadvantage or strain were amplified.

Our findings are broadly consistent with those from HIC, in suggesting an association between disability in older adult residents and household economic disadvantage and strain. Our headline finding of a 12% lower consumption level in chronic care households (based upon count ratios meta-analysed across sites, with no heterogeneity), controlling for baseline household socioeconomic status and household composition, is similar to the cost of disability in Vietnam, amounting to around 9% of household income using the ‘standard of living’ approach (Minh et al., 2015). This, and other studies have emphasized that state benefits fail to compensate for the increased costs of disability and needs for care, even in states with relatively well developed welfare systems (Dushi & Rupp, 2013; Schofield et al., 2013; Cullinan et al., 2013). In our study, total household income from paid work was lower in care households and, as illustrated by qualitative narratives, was probably accounted for by work incapacity in the IOP, as well as working age adult residents forgoing working opportunities to care. However, income from government transfers was also significantly lower in care households. Since, for most of the households where older people live, government transfers were made up largely of pension income (Prince et al., 2016), this probably reflects the lifecourse economic disadvantage of those in informal occupations, and their resulting poor health outcomes in late life. Certainly, in our qualitative study, where pensions existed, although the regularity of the contribution to household income was welcomed, pensions were seen as small, unfairly distributed and insufficient to mitigate costs associated with older age ([Mayston R](#_ENREF_1)). Nevertheless, such inequities could be reduced through social pensions, poverty alleviation cash transfers (e.g. ’70 y Mas’ in Mexico (Salinas-Rodriguez et al., 2014; Prince et al., 2016)), and more targeted benefits in the form of caregiver allowances and disability pensions. Caregiver allowances do not exist in the countries studied, and disability benefits have very minimal coverage. Private transfers of cash or goods from outside of the home also did nothing to decrease the relative economic impact of care dependence, since these were received at a similar level by households with and without care dependent older adults. Qualitative evidence relating to intergenerational transfers suggested that economic contributions from kin outside the household were commonly made according to availability of resources, as demonstration of filial duty rather than a specific response to household needs ([Mayston R](#_ENREF_1)).

In summary, we report some of the first direct and detailed evidence, from middle income countries of a discernable negative economic impact on household-level economic functioning associated with care dependence of older adult residents. While this is a longitudinal study with careful control for previous household economic status we cannot confidently attribute causality, since there may have been some covariance of economic and health disadvantage over the life-course. Nevertheless, given the associated high direct costs of health and social care, and livelihood opportunity costs of caregivers, the associations are highly plausible. The elucidation of these effects at household level is an important finding. The needs of older people have, hitherto, never been prominent in the global health and development agendas. This study emphasizes that the health and wellbeing of older people, living, typically, in multigenerational households, and largely dependent upon their families for their basic needs, is inextricably linked with that of the household unit and extended family (Prince et al., 2008). Population ageing will rapidly increase the numbers of ‘households where older people live’ and their societal significance. Numbers of care dependent older people may quadruple in LMIC through to 2050, while numbers of younger care dependent people remain stable (Harwood, Sayer, & Hirschfeld, 2004). An urgent policy response is needed to make long-term care arrangements sustainable into the future. This is likely to require some combination of improved income security in old age (social pensions, and greater access to contributory schemes), incentivisation of informal care through compensation for direct and opportunity costs (disability benefits and caregiver allowances), and incremental provision of structured social care services to support, and, where necessary, supplement or substitute the central role of informal caregivers.

Funding

This work was supported by DFID/ESRC Joint Fund for Poverty Alleviation Research (RES-167-25-0754 - The Economic and Social Effects of Care Dependence in Later Life); the Wellcome Trust (GR080002- Incidence phase in Peru, Mexico and China); the US Alzheimer’s Association (IIRG-04-1286 - baseline phase in Mexico and Peru); and the World Health Organization (baseline phase in China). The funders had no role in study design, data collection and analysis, decision to publish, or preparation of the manuscript.

Table 1

Original household designation (at the time of selection) and redesignation (upon tracing for INDEP survey)

| Original household designation | Peru urban | Peru rural | Mexico urban | Mexico rural | China urban | China rural | All sites |
| --- | --- | --- | --- | --- | --- | --- | --- |
| No care | 138 | 49 | 123 | 112 | 168 | 82 | 672 |
| Incident care | 87 | 38 | 84 | 87 | 124 | 73 | 493 |
| Chronic care | 51 | 11 | 37 | 25 | 56 | 9 | 189 |
| Total | 276 | 98 | 244 | 224 | 348 | 164 | 1354 |
| Redesignation process^1^ | Peru urban | Peru rural | Mexico urban | Mexico rural | China urban | China rural | All sites |
| No care> No care lost | 21 (15%) | 4  (8%) | 17 (14%) | 13 (12%) | 10  (6%) | 3  (4%) | 68 (10%) |
| Incident care> care exit | 36 (41%) | 10 (26%) | 35 (42%) | 34 (39%) | 51 (41%) | 33 (45%) | 199 (40%) |
| Chronic care> care exit | 23 (45%) | 4  (36%) | 18 (49%) | 12 (48%) | 25 (45%) | 7  (78%) | 89 (47%) |
| Redesignated household categories | Peru urban | Peru rural | Mexico urban | Mexico rural | China urban | China rural | All sites |
| No care | 117 | 45 | 106 | 99 | 158 | 79 | 604 |
| Incident care | 51 | 28 | 49 | 53 | 73 | 40 | 294 |
| Chronic care | 28 | 7 | 19 | 13 | 31 | 2 | 100 |
| Care exit | 59 | 14 | 53 | 46 | 76 | 40 | 288 |
| Total | 255 | 94 | 227 | 211 | 338 | 161 | 1286 |

1. number, and percentage of all those in the original designation

Table 2.

Response proportions for household interview and individual older person interview^1^, at household level, by site

| Site | Interview | Incident care | Chronic care | Care exit | No care | All groups |
| --- | --- | --- | --- | --- | --- | --- |
| China urban | Household | 49/73  (67%) | 15/31  (48%) | 21/76  (28%) | 91/158  (58%) | 176/338  (52%) |
|  | Individual* | All 48/49  None 1/49 | All 15/15 | Not required | All 89/91  Some 1/91  None 1/91 | All 152/155  Some 1/155  None 2/155 |
| China rural | Household | 40/40  (100%) | 2/2  (100%) | 40/40  (100%) | 62/79  (79%) | 144/161  (89%) |
|  | Individual | All 39/40  Some 1/40 | All 2/2 | Not required | All 52/62  Some 10/62 | All 93/104  Some 11/104 |
| Peru urban | Household | 31/51  (61%) | 15/28  (54%) | 22/59  (37%) | 72/117  62%) | 140/255  (55%) |
|  | Individual | All 27/31  Some 3/31  None 1/31 | All 12/15  Some 3/15 | Not required | All 66/72  Some 6/72 | All 105/118  Some 12/118  None 1/31 |
| Peru rural | Household | 17/28  (61%) | 5/7  (71%) | 6/14  (43%) | 28/45  (62%) | 56/94  (60%) |
|  | Individual | All 17/17 | All 5/5 | Not required | All 27/28  Some 1/28 | All 49/50  Some 1/50 |
| Mexico urban | Household | 44/49  (90%) | 18/19  (95%) | 36/53  (68%) | 91/106  (86%) | 189/227  (83%) |
|  | Individual | All 41/44  Some 3/44 | All 15/18  Some 3/18 | Not required | All 85/91  Some 6/91 | All 141/153  Some 12/153 |
| Mexico rural | Household | 46/53  (87%) | 12/13  (92%) | 29/46  (63%) | 80/99  (81%) | 167/211  (79%) |
|  | Individual | All 44/46  Some 2/46 | All 11/12  Some 1/12 | Not required | All 76/80  Some 3/80  None 1/80 | All 131/138  Some 6/138  None 1/138 |
| All sites | Household | 227/294  (77%) | 67/100  (67%) | 154/288  (54%) | 424/604  (70%) | 872/1286  (68%) |
|  | Individual | All 216/227  Some 9/227  None 2/227 | All 60/67  Some 7/67 | Not required | All 395/424  Some 27/424  None 2/424 | All 671/718  Some 43/718  None 4/718 |

1. Profile of individual interviews at household level, that is the number of households at which all eligible index older people (all), some eligibles (some) or none of those eligible (none) were interviewed. An IOP was eligible for interview if they were alive and still resident at the household.

Table 3

Sociodemographic characteristics of households completing household interview at time of selection and at INDEP interview^1^, and associations with household care status (weighted analysis^2^)

|  | Peru urban | Peru rural | Mexico urban | Mexico rural | China urban | China rural | All sites | Association (PR) with household care status (incident and chronic care vs no care) |
| --- | --- | --- | --- | --- | --- | --- | --- | --- |
| Number of households (weighted number) | 140 (705) | 56 (371) | 189 (620) | 167 (610) | 176 (508) | 144 (587) | 872 (3401) |  |
| At household selection |  |  |  |  |  |  |  |  |
| Mean number of residents (SD) | 4.4 (2.1) | 4.0 (2.4) | 4.0 (2.8) | 3.4 (1.9) | 2.8 (1.2) | 3.9 (1.7) | 3.7 (1.7) | 1.04 (0.99-1.08) |
| Co-resident children aged <16 years (%) | 52.1 | 41.8 | 34.2 | 27.0 | 8.3 | 22.8 | 31.6 | 1.07 (0.87-1.31) |
| Index older person’s (IOP) living arrangements |  |  |  |  |  |  |  |  |
| Alone (%) | 8.0 | 14.6 | 14.7 | 8.0 | 5.5 | 7.2 | 9.4 | 1 (ref) |
| With spouse only (%) | 10.5 | 4.9 | 6.6 | 12.8 | 30.5 | 21.3 | 14.4 | 1.73 (1.06-2.84) |
| With adult children +/- others (%) | 58.4 | 63.3 | 63.9 | 65.6 | 48.8 | 59.3 | 60.0 | 1.70 (1.08-2.68) |
| Other arrangement (%) | 23.2 | 17.3 | 17.3 | 13.6 | 15.2 | 12.3 | 16.2 | 1.78 (1.10-2.90) |
| Mean assets (SD)^3^ | 6.2 (0.5) | 4.9 (1.0) | 6.0 (1.0) | 4.0 (1.8) | 5.4 (0.6) | 5.5 (1.3) | 5.4 (1.4) | 0.99 (0.93-1.06) |
| Highest occupational status among IOPs (skilled or manual labourer %) | 27.9 | 91.3 | 60.4 | 91.3 | 42.4 | 96.1 | 66.1 | 1.04 (0.96-1.13)^4^ |
| At INDEP interview |  |  |  |  |  |  |  |  |
| Household change (%) | 1.8 | 2.2 | 2.4 | 13.7 | 5.2 | 35.1 | 8.9 | 1.12 (0.83-1.52) |
| Mean number of residents | 4.4 (2.4) | 4.3 (2.4) | 3.3 (1.9) | 3.8 (2.3) | 2.6 (1.3) | 4.1 (1.6) | 3.7 (2.1) | 1.01 (0.97-1.06) |
| Mean change in number of residents from baseline | -0.1 (2.0) | +0.3 (1.7) | -0.7 (3.0) | +0.5 (2.8) | -0.3 (1.1) | +0.3 (1.8) | 0.0 (2.3) | 0.98 (0.94-1.02) |
| Mean assets (SD)^3^ | 9.1 (1.3) | 7.2 (2.5) | 8.2 (1.4) | 6.4 (1.8) | 8.4 (1.5) | 8.6 (1.9) | 8.0 (2.0) | 0.99 (0.94-1.04) |

1. Households were selected from the incidence wave of the 10/66 survey, and data on household characteristics were collected at that time. Recontacting for INDEP interviews was carried out three years later

2. Weighted for sampling fraction, and response

3. An extended assets scale was used for the INDEP survey, and 10/66 survey and INDEP survey assets data are therefore not directly comparable

4. Per occupational status level

Table 4. Associations between redesignated household status (no care versus incident care, chronic care and care exit households) and main household economic welfare indicators (income, consumption, strain and satisfaction)

| **Equivalised**^1^ **household income**  Negative binomial regression – adjusted^2^ count ratios | Site/ Country | No care  n=424 | Current care  n=292 | Incident care  n=225 | Chronic care  n=67 | Care exit  n=156 |
| --- | --- | --- | --- | --- | --- | --- |
|  | Peru urban | 1 (ref) | 1.01 (0.86-1.18) | 0.99 (0.83-1.19) | 1.03 (0.82-1.30) | 1.01 (0.82-1.23) |
|  | Peru rural | 1 (ref) | 0.84 (0.58-1.22) | 0.83 (0.55-1.25) | 1.05 (0.52-2.13) | 1.00 (0.55-1.83) |
|  | Mexico urban | 1 (ref) | 0.99 (0.81-1.21) | 1.03 (0.83-1.30) | 0.84 (0.61-1.15) | 1.04 (0.80-1.34) |
|  | Mexico rural | 1 (ref) | 0.88 (0.68-1.15) | 0.92 (0.68-1.23) | 0.84 (0.51-1.38) | 1.09 (0.76-1.56) |
|  | China urban | 1 (ref) | 1.45 (1.07-1.96) | **1.61 (1.17-2.21)** | 1.03 (0.64-1.66) | **0.63 (0.42-0.94)** |
|  | China rural | 1 (ref) | 0.78 (0.49-1.23) | 0.79 (0.49-1.29) | **0.19 (0.04-0.99)** | **1.67 (1.04-2.67)** |
|  | Pooled CR | 1 (ref) | 1.00 (0.91-1.10) | 1.02 (0.92-1.14) | 0.94 (0.81-1.11) | 1.02 (0.89-1.16) |
|  | I squared |  | 42.5% | 52.5% | 6.3% | 49.3% |
| **Household income from paid work (controlling also for number of adults)**  Zero inflated negative binomial regression – adjusted^2^ count ratios | Site/ Country | No care | Current care | Incident care | Chronic care | Care exit |
|  | Peru urban | 1 (ref) | 0.82 (0.66-1.03) | 0.87 (0.67-1.13) | 0.79 (0.57-1.10) | 1.01 (0.77-1.33) |
|  | Peru rural | 1 (ref) | 1.06 (0.72-1.55) | 1.14 (0.40-2.01) | 0.90 (0.40-2.01) | 1.64 (0.78-3.44) |
|  | Mexico urban | 1 (ref) | 1.17 (0.83-1.67) | 1.36 (0.93-1.99) | 0.92 (0.60-1.40) | 1.10 (0.73-1.63) |
|  | Mexico rural | 1 (ref) | 1.19 (0.77-1.84) | 1.34 (0.82-2.18) | 1.05 (0.46-2.42) | 1.33 (0.84-2.10) |
|  | China urban | 1 (ref) | **0.63 (0.42-0.94)** | **0.56 (0.35-0.90)** | 1.75 (0.58-5.26) | 0.34 (0.16-0.73) |
|  | China rural | 1 (ref) | 0.79 (0.61-1.02) | **0.78 (0.61-0.99)** | 1.12 (0.40-3.13) | 0.99 (0.78-1.27) |
|  | Pooled CR | 1 (ref) | **0.88 (0.78-1.00)** | 0.90 (0.78-1.04) | 0.90 (0.71-1.12) | 1.02 (0.88-1.19) |
|  | I squared |  | 44.5% | 60.6% | 0.0% | 55.0% |
| **Equivalised household income from private transfers**  Zero inflated negative binomial regression – adjusted^2^ count ratios | Site/ Country | No care | Current care | Incident care | Chronic care | Care exit |
|  | Peru urban | 1 (ref) | 0.96 (0.67-1.38) | 0.85 (0.55-1.32) | 1.18 (0.69-2.02) | 1.20 (0.63-2.31) |
|  | Peru rural | 1 (ref) | 0.98 (0.48-2.00) | 0.99 (0.48-2.03) | None with income | None with income |
|  | Mexico urban | 1 (ref) | 1.12 (0.91-1.39) | 1.16 (0.92-1.47) | 0.97 (0.68-1.38) | 0.82 (0.58-1.16) |
|  | Mexico rural | 1 (ref) | 0.75 (0.29-1.98) | 0.76 (0.30-1.92) | None with income | 0.84 (0.19-3.71) |
|  | China urban | 1 (ref) | 1.13 (0.68-1.87) | 1.34 (0.80-2.25) | 0.54 (0.26-1.14) | 0.51 (0.25-1.07) |
|  | China rural | 1 (ref) | 0.59 (0.25-1.39) | 0.64 (0.26-1.57) | 0.40 (0.04-3.70) | 0.38 (0.08-1.70) |
|  | Pooled CR | 1 (ref) | 1.04 (0.89-1.23) | 1.07 (0.89-1.28) | 0.93 (0.71-1.22) | 0.80 (0.61-1.05) |
|  | I squared |  | 0.0% | 0.0% | 12.3% | 0.0% |
| **Equivalised household income from government transfers**  Zero inflated negative binomial regression – adjusted^2^ count ratios | Site/ Country | No care | Current care | Incident care | Chronic care | Care exit |
|  | Peru urban | 1 (ref) | 2.45 (0.81-7.42) | 1.18 (0.35-4.07) | None with income | 1.32 (0.53-3.33) |
|  | Peru rural | 1 (ref) | None with income | None with income | None with income | None with income |
|  | Mexico urban | 1 (ref) | **0.79 (0.67-0.94)** | **0.80 (0.66-0.97)** | 0.78 (0.59-1.05) | 0.80 (0.61-1.07) |
|  | Mexico rural | 1 (ref) | 1.21 (0.73-2.02) | 0.92 (0.30-2.80) | 0.47 (0.19-1.16) | 0.88 (0.40-1.92) |
|  | China urban | 1 (ref) | **0.54 (0.35-0.85)** | **0.53 (0.32-0.89)** | 0.63 (0.31-1.29) | 5.43 (2.58-11.42) |
|  | China rural | 1 (ref) | 0.65 (0.18-2.37) | 0.83 (0.26-2.60) | 0.13 (0.01-2.74) | 0.78 (0.23-2.62) |
|  | Pooled CR | 1 (ref) | **0.80 (0.69-0.93)** | **0.77 (0.65-0.92)** | **0.72 (0.56-0.93)** | 1.01 (0.80-1.28) |
|  | I squared |  | 58.3% | 0.0% | 0.0% | 82.5% |
| **Equivalised household consumption**  Negative binomial regression – adjusted^2^ count ratios |  | No care | Current care | Incident care | Chronic care | Care exit |
|  | Peru urban | 1 (ref) | 0.97 (0.84-1.12) | 1.03 (0.88-1.21) | 0.82 (0.67-1.01) | 1.05 (0.88-1.26) |
|  | Peru rural | 1 (ref) | **0.71 (0.52-0.97)** | **0.67 (0.48-0.94)** | 0.96 (0.55-1.68) | 1.06 (0.64-1.77) |
|  | Mexico urban | 1 (ref) | 1.06 (0.91-1.24) | 1.11 (0.93-1.32) | 0.92 (0.72-1.17) | 0.95 (0.78-1.16) |
|  | Mexico rural | 1 (ref) | 0.94 (0.77-1.14) | 0.91 (0.74-1.12) | 1.06 (0.75-1.49) | 1.05 (0.83-1.33) |
|  | China urban | 1 (ref) | 0.99 (0.84-1.16) | 1.05 (0.88-1.26) | 0.78 (0.59-1.03) | 0.98 (0.77-1.24) |
|  | China rural | 1 (ref) | 0.76 (0.57-1.03) | **0.76 (0.57-1.00)** | 1.03 (0.44-2.44) | 1.05 (0.79-1.40) |
|  | Pooled CR | 1 (ref) | 0.96 (0.89-1.03) | 0.98 (0.90-1.06) | **0.88 (0.77-0.99)** | 1.01 (0.92-1.12) |
|  | I squared |  | 35.6% | 56.3% | 0% | 0% |
| **Equivalised household food consumption**  Negative binomial regression – adjusted^2^ count ratios | Site/ Country | No care | Current care | Incident care | Chronic care | Care exit |
|  | Peru urban | 1 (ref) | 0.92 (0.80-1.06) | 0.96 (0.81-1.14) | 0.82 (0.66-1.01) | 1.07 (0.89-1.29) |
|  | Peru rural | 1 (ref) | 0.91 (0.70-1.19) | 0.87 (0.65-1.16) | 1.12 (0.69-1.80) | 1.20 (0.78-1.85) |
|  | Mexico urban | 1 (ref) | 1.01 (0.83-1.22) | 1.11 (0.90-1.38) | 0.75 (0.56-1.02) | 0.97 (0.76-1.24) |
|  | Mexico rural | 1 (ref) | 1.19 (0.96-1.47) | 1.16 (0.92-1.46) | 1.38 (0.95-1.99) | 1.16 (0.90-1.51) |
|  | China urban | 1 (ref) | 1.01 (0.85-1.21) | 1.07 (0.87-1.30) | 0.84 (0.62-1.13) | 0.97 (0.75-1.25) |
|  | China rural | 1 (ref) | 0.91 (0.63-1.30) | 0.84 (0.61-1.17) | 2.38 (0.88-6.46) | 1.08 (0.77-1.51) |
|  | Pooled CR | 1 (ref) | 0.98 (0.91-1.07) | 1.02 (0.93-1.11) | 0.90 (0.79-1.03) | 1.06 (0.95-1.17) |
|  | I squared |  | 0.0% | 0.3% | 58.1% | 0.0% |
| **Economic strain indicators – last three years**  Ordinal regression – adjusted^2^ odds ratios | Site/ Country | No care | Current care | Incident care | Chronic care | Care exit |
|  | Peru urban | 1 (ref) | **2.46 (1.15-5.27)** | 1.85 (0.79-4.38) | **3.70 (1.28-10.72)** | 2.30 (0.84-6.30) |
|  | Peru rural | 1 (ref) | 3.18 (0.86-11.68) | **4.14 (1.08-15.85)** | 0.88 (0.10-7.95) | 2.83 (0.42-19.32) |
|  | Mexico urban | 1 (ref) | 0.72 (0.39-1.36) | 0.73 (0.37-1.48) | 0.64 (0.23-1.80) | 0.66 (0.31-1.40) |
|  | Mexico rural | 1 (ref) | 1.43 (0.73-2.80) | 1.66 (0.82-3.39) | 0.83 (0.23-3.04) | 1.62 (0.70-3.74) |
|  | China urban | 1 (ref) | 2.59 (0.86-7.86) | 2.17 (0.62-7.57) | 3.91 (0.89-17.19) | 0.77 (0.08-6.95) |
|  | China rural | 1 (ref) | 0.63 (0.18-2.20) | 0.51 (0.14-1.90) | 16.86 (0.50-566.55) | 0.69 (0.21-2.28) |
|  | Pooled OR | 1 (ref) | 1.37 (0.97-1.92) | 1.33 (0.92-1.94) | 1.58 (0.90-2.77) | 1.15 (0.75-1.77) |
|  | I squared |  | 53.3% | 45.8% | 49.2% | 19.7% |
| **Dissatisfaction with economic circumstances**  Ordinal regression – adjusted^2^ odds ratios | Site/ Country | No care | Current care | Incident care | Chronic care | Care exit |
|  | Peru urban | 1 (ref) | 1.48 (0.68-3.24) | 1.09 (0.45-2.62) | 2.02 (0.66-6.13) | 1.20 (0.44-3.28) |
|  | Peru rural | 1 (ref) | **4.19 (1.18-14.85)** | **6.89 (1.77-26.81)** | 1.00 (0.13-7.74) | **7.05 (1.00-49.62)** |
|  | Mexico urban | 1 (ref) | 0.91 (0.49-1.69) | 0.96 (0.48-1.93) | 0.89 (0.34-2.28) | 0.73 (0.35-1.51) |
|  | Mexico rural | 1 (ref) | 0.78 (0.37-1.63) | 0.85 (0.40-1.83) | 0.72 (0.22-2.39) | **0.37 (0.15-0.93)** |
|  | China urban | 1 (ref) | **2.23 (1.10-4.49)** | 1.29 (0.58-2.80) | **13.79 (3.80-50.01)** | 0.95 (0.33-2.74) |
|  | China rural | 1 (ref) | 0.81 (0.24-2.74) | 0.57 (0.17-1.94) | 8.12 (0.27-247.58) | 0.38 (0.10-1.42) |
|  | Pooled OR | 1 (ref) | 1.28 (0.92-1.77) | 1.11 (0.78-1.58) | **1.74 (1.02-2.97)** | 0.70 (0.46-1.06) |
|  | I squared |  | 46.3% | 43.6% | 66.8% | 57.7% |

Notes

1. All estimates are controlled for household assets at baseline, occupational class (highest among older people at baseline), and household composition at baseline (older person alone, with spouse only, with other adults, with other adults and children)

2. Equivalised income and consumption is total household income or consumption adjusted for household size, by dividing by (1 + (0.5 x number of adults beyond 1) + (0.3 x number of children))

Table 5. Associations between household status (no care vs incident care, chronic care and care exit) and out of pocket healthcare expenditure, catastrophic healthcare spending, and not engaging in education or paid work to care for an older adult

| **Household healthcare expenditure**  Zero inflated negative binomial regression – adjusted^1^ count ratios |  | No care  n=424 | Current care  n=292 | Incident  n=225 | Chronic  n=67 | Care exit  n=156 |
| --- | --- | --- | --- | --- | --- | --- |
|  | Peru urban | 1 (ref) | **2.32 (1.68-3.20)** | **2.08 (1.46-2.97)** | **2.14 (1.43-3.20)** | 0.68 (0.42-1.11) |
|  | Peru rural | 1 (ref) | 0.79 (0.27-2.35) | **0.46 (0.25-0.84)** | 1.21 (0.45-3.28) | **0.07 (0.03-0.17)** |
|  | Mexico urban | 1 (ref) | 0.86 (0.50-1.47) | 0.94 (0.51-1.73) | 0.64 (0.27-1.51) | 0.68 (0.30-1.51) |
|  | Mexico rural | 1 (ref) | 0.79 (0.46-1.37) | 0.63 (0.35-1.12) | 1.39 (0.52-3.76) | 0.48 (0.19-1.18) |
|  | China urban | 1 (ref) | **1.87 (1.23-2.83)** | **1.99 (1.27-3.11)** | 0.68 (0.33-1.41) | 1.39 (0.67-2.87) |
|  | China rural | 1 (ref) | 1.37 (0.52-3.59) | 2.19 (0.72-6.61) | 0.78 (0.05-13.50) | **0.06 (0.02-0.16)** |
|  | Pooled CR | 1 (ref) | **1.55 (1.26-1.90)** | **1.33 (1.07-1.64)** | **1.40 (1.04-1.87)** | **0.46 (0.34-0.62)** |
|  | I squared |  | 73.4% | 83.0% | 56.5% | 88.9% |
| **Catastrophic healthcare expenditure****  Poisson regression – adjusted^1^ relative risks |  | No care | Current care | Incident care | Chronic care | Care exit |
|  | Peru urban | 1 (ref) | **4.88 (1.73-13.79)** | **4.48 (1.46-13.73)** | **5.67 (1.66-19.34)** | None with outcome |
|  | Peru rural | 1 (ref) | 2.36 (0.53-10.63) | 2.34 (0.48-11.34) | 11.29 (0.30-419.71) | None with outcome |
|  | Mexico urban | 1 (ref) | 1.55 (0.85-2.85) | 1.72 (0.90-3.27) | 1.17 (0.44-3.14) | 0.46 (0.13-1.58) |
|  | Mexico rural | 1 (ref) | 1.14 (0.61-2.15) | 1.28 (0.66-2.49) | 0.75 (0.22-2.60) | 0.60 (0.20-1.77) |
|  | China urban | 1 (ref) | 1.70 (0.94-3.11) | 1.79 (0.95-3.40) | 1.44 (0.52-3.99) | 1.06 (0.42-2.64) |
|  | China rural | 1 (ref) | 1.36 (0.57-3.27) | 1.30 (0.53-3.19) | 2.21 (0.38-17.61) | 0.14 (0.02-1.08) |
|  | Pooled PR | 1 (ref) | **1.64 (1.20-2.22)** | **1.71 (1.24-2.37)** | 1.67 (0.99-2.81) | 0.63 (0.35-1.13) |
|  | I squared |  | 16.3% | 0% | 30.2% | 18.2% |
| **Another resident is not working (main reason cited is to care for older person)**  Poisson regression – adjusted^2^ relative risks |  | No care | Current care | Incident care | Chronic care | Care exit |
|  | Peru urban | 1 (ref) | 2.05 (0.85-4.94) | 2.31 (0.90-5.92) | 1.53 (0.41-5.70) | Omitted |
|  | Peru rural | 1 (ref) | **5.98 (1.36-26.18)** | **7.01 (1.52-32.45)** | 2.15 (0.15-30.61) | Omitted |
|  | Mexico urban | 1 (ref) | **5.12 (1.87-14.01)** | **5.50 (1.94-15.60)** | **4.18 (1.11-15.73)** | Omitted |
|  | Mexico rural | 1 (ref) | 1.35 (0.52-3.49) | 1.38 (0.50-3.79) | 1.27 (0.25-6.40) | Omitted |
|  | China urban | 1 (ref) | 0.90 (0.04-18.91) | None exposed | 11.65 (0.02-5699.85) | Omitted |
|  | China rural | 1 (ref) | 1.36 (0.65-2.84) | 1.33 (0.63-2.81) | 1.95 (0.25-15.20) | Omitted |
|  | Pooled PR | 1 (ref) | **2.08 (1.37-3.16)** | **2.22 (1.43-3.43)** | **2.15 (1.04-4.42)** | Omitted |
|  | I squared |  | 32.3% | 48.3% | 0% |  |

1. Controlled for household assets at baseline, occupational class (highest among older people at baseline), and household composition at baseline (older person alone, with spouse only, with other adults, with other adults and children), and number of adult and number of child residents

2. Controlled for household assets at baseline, occupational class (highest among older people at baseline), and household composition at baseline (older person alone, with spouse only, with other adults, with other adults and children)

Online Table 1

Sensitivity analysis (excluding household changes, and no care households where one or more older residents have developed needs for care). Associations between redesignated household status (no care versus incident care, chronic care and care exit households) and main household economic welfare indicators (income, consumption, strain and satisfaction)

| **Equivalised Household income**  Negative binomial regression – adjusted count ratios | Site/ Country | No care  n=260 | All care  n=254 | Incident  n=192 | Chronic  n=62 | Care exit  n=91 |
| --- | --- | --- | --- | --- | --- | --- |
|  | Peru urban | 1 (ref) | 1.07 (0.91-1.27) | 1.06 (0.88-1.28) | 1.09 (0.86-1.38) | 1.05 (0.85-1.29) |
|  | Peru rural | 1 (ref) | 0.83 (0.55-1.25) | 0.81 (0.52-1.28) | 1.10 (0.49-2.48) | 1.00 (0.52-1.88) |
|  | Mexico urban | 1 (ref) | 1.01 (0.81-1.26) | 1.04 (0.82-1.33) | 0.87 (0.62-1.22) | 1.11 (0.78-1.58) |
|  | Mexico rural | 1 (ref) | 0.97 (0.72-1.31) | 1.02 (0.73-1.41) | 0.86 (0.51-1.45) | 1.09 (0.62-1.92) |
|  | China urban | 1 (ref) | **1.62 (1.16-2.26)** | **1.84 (1.30-2.60)** | 1.09 (0.67-1.75) | 0.73 (0.48-1.12) |
|  | China rural | 1 (ref) | 0.49 (0.24-1.02) | 0.60 (0.28-1.28) | 0.22 (0.02-2.02) | 1.94 (0.84-4.46) |
|  | Pooled CR | 1 (ref) | 1.05 (0.94-1.17) | **1.20 (1.09-1.33)** | 1.00 (0.85-1.18) | 1.03 (0.89-1.20) |
|  | I squared |  | 59.9% | 88.4% | 0.0% | 0.0% |
| **Equivalised Household expenditure**  Negative binomial regression – adjusted count ratios | Site/ Country | No care | All care | Incident | Chronic | Care exit |
|  | Peru urban | 1 (ref) | 0.96 (0.82-1.12) | 1.01 (0.85-1.21) | 0.82 (0.66-1.03) | 1.06 (0.88-1.29) |
|  | Peru rural | 1 (ref) | **0.65 (0.47-0.89)** | **0.61 (0.48-0.87)** | 0.89 (0.48-1.66) | 1.00 (0.60-1.68) |
|  | Mexico urban | 1 (ref) | 1.09 (0.92-1.29) | 1.14 (0.95-1.37) | 0.94 (0.73-1.22) | 1.06 (0.82-1.37) |
|  | Mexico rural | 1 (ref) | 0.98 (0.79-1.21) | 0.96 (0.77-1.21) | 1.04 (0.72-1.48) | 1.05 (0.71-1.55) |
|  | China urban | 1 (ref) | 0.94 (0.78-1.12) | 1.00 (0.82-1.22) | **0.74 (0.55-0.99)** | 0.93 (0.72-1.19) |
|  | China rural | 1 (ref) | **0.47 (0.31-0.72)** | **0.51 (0.34-0.75)** | 0.43 (0.13-1.37) | 1.05 (0.79-1.40) |
|  | Pooled CR | 1 (ref) | 0.94 (0.86-1.02) | 0.95 (0.87-1.04) | **0.86 (0.75-0.98)** | 1.03 (0.92-1.15) |
|  | I squared |  | 73.2% | 77.8% | 0.0% | 0.0% |
| **Equivalised food consumption**  Negative binomial regression – adjusted count ratios | Site/ Country | No care | All care | Incident | Chronic | Care exit |
|  | Peru urban | 1 (ref) | 0.89 (0.77-1.03) | 0.92 (0.77-1.09) | 0.83 (0.66-1.03) | 1.05 (0.87-1.28) |
|  | Peru rural | 1 (ref) | 0.88 (0.66-1.18) | 0.84 (0.62-1.15) | 1.12 (0.66-1.91) | 1.17 (0.75-1.82) |
|  | Mexico urban | 1 (ref) | 1.04 (0.84-1.28) | 1.14 (0.90-1.43) | 0.78 (0.57-1.06) | 1.00 (0.73-1.37) |
|  | Mexico rural | 1 (ref) | 1.26 (0.99-1.60) | 1.25 (0.97-1.62) | 1.29 (0.87-1.93) | 0.99 (0.63-1.57) |
|  | China urban | 1 (ref) | 1.02 (0.83-1.24) | 1.07 (0.87-1.32) | 0.86 (0.63-1.17) | 0.97 (0.74-1.26) |
|  | China rural | 1 (ref) | 0.70 (0.41-1.19) | 0.66 (0.41-1.08) | 1.88 (0.45-7.92) | 1.09 (0.62-1.91) |
|  | Pooled CR | 1 (ref) | 0.98 (0.90-1.07) | 1.01 (0.92-1.11) | 0.90 (0.78-1.03) | 1.03 (0.91-1.17) |
|  | I squared |  | 40.6% | 47.0% | 19.5% | 0.0% |
| **Strain in the past three years**  Ordinal regression – adjusted odds ratios | Site/ Country | No care | All care | Incident | Chronic | Care exit |
|  | Peru urban | 1 (ref) | 2.14 (0.93-4.89) | 1.42 (0.56-3.60) | **3.90 (1.25-12.2)** | 2.29 (0.79-6.68) |
|  | Peru rural | 1 (ref) | 3.32 (0.83-13.3) | **4.28 (1.03-17.7)** | 0.67 (0.06-8.00) | 2.90 (0.41-20.4) |
|  | Mexico urban | 1 (ref) | 0.95 (0.48-1.89) | 0.93 (0.44-1.98) | 0.95 (0.33-2.76) | 0.99 (0.34-2.82) |
|  | Mexico rural | 1 (ref) | **2.66 (1.20-5.91)** | **3.10 (1.33-7.20)** | 1.61 (0.40-6.43) | 1.56 (0.98-7.01) |
|  | China urban | 1 (ref) | 4.64 (0.93-23.06) | 3.34 (0.59-18.76) | **9.08 (1.36-60.54)** | 1.51 (0.13-18.0) |
|  | China rural | 1 (ref) | 0.21 (0.04-1.12) | **0.15 (0.02-0.91)** | 4.36 (0.10-191.7) | 0.43 (0.05-4.83) |
|  | Pooled CR | 1 (ref) | **1.64 (1.11-2.44)** | **1.58 (1.03-2.42)** | **2.03 (1.10-3.73)** | 1.49 (0.87-2.56) |
|  | I squared |  | 60.6% | 61.1% | 25.1% | 0.0% |
| **Satisfaction with economic circumstances**  Ordinal regression – adjusted odds ratios | Site/ Country | No care |  |  |  |  |
|  | Peru urban | 1 (ref) | 1.83 (0.78-4.34) | 1.23 (0.48-3.13) | 2.60 (0.80-8.43) | 1.46 (0.51-4.22) |
|  | Peru rural | 1 (ref) | 3.14 (0.81-12.06) | 6.26 (1.49-26.32) | 0.65 (0.07-6.17) | 5.88 (0.83-41.67) |
|  | Mexico urban | 1 (ref) | 0.91 (0.46-1.80) | 0.95 (0.45-1.99) | 1.07 (0.40-2.87) | 0.73 (0.26-2.01) |
|  | Mexico rural | 1 (ref) | 0.93 (0.40-2.13) | 1.06 (0.43-2.58) | 0.64 (0.17-2.49) | 0.10 (0.02-0.52) |
|  | China urban | 1 (ref) | **4.38 (1.81-10.57)** | 2.54 (0.98-6.58) | 28.05 (6.87-114.44) | 1.99 (0.62-6.46) |
|  | China rural | 1 (ref) | 0.78 (0.17-3.69) | 0.68 (0.13-3.41) | DNC | 0.87 (0.61-52.90) |
|  | Pooled CR | 1 (ref) | **1.50 (1.04-2.18)** | 1.38 (0.92-2.07) | **2.02 (1.13-3.59)** | 1.03 (0.60-1.77) |
|  | I squared |  | 54.7% | 36.9% | 79.0% | 61.5% |

Notes

1. All estimates are controlled for household assets at baseline, occupational class (highest among older people at baseline), and household composition at baseline (older person alone, with spouse only, with other adults, with other adults and children)

2. Equivalised income and consumption is total household income or consumption adjusted for household size, by dividing by (1 + (0.5 x number of adults beyond 1) + (0.3 x number of children))

Acosta, D., Rottbeck, R., Rodriguez, G., Ferri, C. P., & Prince, M. J. (2008). The epidemiology of dependency among urban-dwelling older people in the Dominican Republic; a cross-sectional survey. *BMC.Public Health, 8,* 285.

Agahi, N., Shaw, B. A., & Fors, S. (2014). Social and economic conditions in childhood and the progression of functional health problems from midlife into old age. *J.Epidemiol.Community Health., 68,* 734-740.

Cullinan, J., Gannon, B., & O'Shea, E. (2013). The welfare implications of disability for older people in Ireland. *Eur.J.Health Econ., 14,* 171-183.

Dushi, I. & Rupp, K. (2013). Disability shocks near retirement age and financial well-being. *Soc.Secur.Bull., 73,* 23-43.

Fonareva, I. & Oken, B. S. (2014). Physiological and functional consequences of caregiving for relatives with dementia. *Int.Psychogeriatr., 26,* 725-747.

Groce, N., Kembhavi, G., Wirz, S., Lang, R., Trani, J.-F., & Kett, M. (2011). *Poverty and disability - a critical review of the literature in Low and Middle-Income Countries* (Rep. No. 16). London: Leonard Cheshire Disability and Inculsive Development Centre.

Guerra, R. O., Alvarado, B. E., & Zunzunegui, M. V. (2008). Life course, gender and ethnic inequalities in functional disability in a Brazilian urban elderly population. *Aging Clin.Exp.Res., 20,* 53-61.

Gureje, O., Ogunniyi, A., Kola, L., & Afolabi, E. (2006). Functional disability in elderly Nigerians: Results from the Ibadan Study of Aging. *J.Am.Geriatr.Soc., 54,* 1784-1789.

Harwood, R. H., Sayer, A. A., & Hirschfeld, M. (2004). Current and future worldwide prevalence of dependency, its relationship to total population, and dependency ratios. *Bull.World Health Organ., 82,* 251-258.

Hosseinpoor, A. R., Stewart Williams, J. A., Gautam, J., Posarac, A., Officer, A., Verdes, E. et al. (2013). Socioeconomic inequality in disability among adults: a multicountry study using the World Health Survey. *Am.J.Public Health., 103,* 1278-1286.

Mayston, R., Guerra, M., Huang, Y., Sosa, A. L., Uwakwe, R., Acosta, I. et al. (2014). Exploring the economic and social effects of care dependence in later life: protocol for the 10/66 research group INDEP study. *Springerplus., 3:379. doi: 10.1186/2193-1801-3-379. eCollection;%2014.,* 379-3.

Mayston R, L.-S. P., Gallardo S, Wang H, Montes de Oca V, Ezeah P, Guerra M, Sosa AL, Uwakwe R, Guerchet M, Prince M. A journey without maps- balancing the costs of caring for dependent older people in Nigeria, China, Mexico and Peru. *In preparation*.

Minh, H. V., Giang, K. B., Liem, N. T., Palmer, M., Thao, N. P., & Duong, l. B. (2015). Estimating the extra cost of living with disability in Vietnam. *Glob.Public Health., 10 Supppl 1:S70-9. doi: 10.1080/17441692.2014.971332. Epub;%2014 Oct 29.,* S70-S79.

Prince, M. (2004). Care arrangements for people with dementia in developing countries. *Int.J.Geriatr.Psychiatry, 19,* 170-177.

Prince, M., Acosta, D., Albanese, E., Arizaga, R., Ferri, C. P., Guerra, M. et al. (2008). Ageing and dementia in low and middle income countries-Using research to engage with public and policy makers. *Int.Rev.Psychiatry, 20,* 332-343.

Prince, M., Brodaty, H., Uwakwe, R., Acosta, D., Ferri, C. P., Guerra, M. et al. (2012). Strain and its correlates among carers of people with dementia in low-income and middle-income countries. A 10/66 Dementia Research Group population-based survey. *Int J Geriatr.Psychiatry, 27,* 670-682.

Prince, M., Ferri, C. P., Acosta, D., Albanese, E., Arizaga, R., Dewey, M. et al. (2007). The protocols for the 10/66 Dementia Research Group population-based research programme. *BMC.Public Health., 7,* 165.

Prince, M. J., Lloyd-sherlock, P., Guerra, M., Huang, Y., Sosa, A. L., Uwakwe, R. et al. (2016). The economic status of older peoples households in urban and rural settings in Peru, Mexico and China: a 10/66 INDEP study cross-sectional survey. *SpringerPlus, 5,* 1-16.

Salinas-Rodriguez, A., Manrique-Espinoza, B., Moreno-Tamayo, K., Torres-Pereda, P., De la Cruz-Gongora, V., Angeles-Tagliaferro, G. et al. (2014). *Impact evaluation of the non-contributory social pension programme 70 y mas in Mexico* (Rep. No. 3ie Impact Evaluation Report 5). New Delhi: International Initiative for Impact Evaluation (3ie).

Schofield, D. J., Callander, E. J., Shrestha, R. N., Percival, R., Kelly, S. J., & Passey, M. E. (2013). Premature retirement due to ill health and income poverty: a cross-sectional study of older workers. *BMJ Open., 3,* e002683.

Sousa, R. M., Ferri, C. P., Acosta, D., Albanese, E., Guerra, M., Huang, Y. et al. (2009). Contribution of chronic diseases to disability in elderly people in countries with low and middle incomes: a 10/66 Dementia Research Group population-based survey. *Lancet, 374,* 1821-1830.

Sousa, R. M., Ferri, C. P., Acosta, D., Guerra, M., Huang, Y., Ks, J. et al. (2010). The contribution of chronic diseases to the prevalence of dependence among older people in Latin America, China and India: a 10/66 Dementia Research Group population-based survey. *BMC.Geriatr., 10,* 53.

Tampubolon, G. (2015). Growing Up in Poverty, Growing Old in Infirmity: The Long Arm of Childhood Conditions in Great Britain. *PLoS.ONE., 10,* e0144722.

Teerawichitchainan, B. & Knodel, J. (2015). Economic Status and Old-Age Health in Poverty-Stricken Myanmar. *J.Aging Health., 27,* 1462-1484.

The World Bank (2014). *Purchasing Power Parities and Real Expenditures of World Economies. Summary of Results and Findings of the 2011 International Comparison Program* Washington DC: International Bank for Reconstruction and Development/ The World Bank.

Uwakwe, R., Ibeh, C. C., Modebe, A. I., Bo, E., Ezeama, N., Njelita, I. et al. (2009). The Epidemiology of Dependence in Older People in Nigeria: Prevalence, Determinants, Informal Care, and Health Service Utilization. A 10/66 Dementia Research Group Cross-Sectional Survey. *J.Am.Geriatr.Soc.*.

Zimmer, Z. (2008). Poverty, wealth inequality and health among older adults in rural Cambodia. *Soc.Sci.Med., 66,* 57-71.

Zunzunegui, M. V., Alvarado, B. E., Beland, F., & Vissandjee, B. (2009). Explaining health differences between men and women in later life: a cross-city comparison in Latin America and the Caribbean. *Soc.Sci.Med., 68,* 235-242.

**ANNEX - Details of the methodology**

In this annex we provide further contextual information on

1. The catchment area sites in Peru, Mexico and China selected for the 10/66 population-based surveys and the nested INDEP cohort study, and the local and national changes that have impacted since the beginning of the 10/66 Dementia Research Group (10/66 DRG) studies in 2003-2006.

2. The outcomes measures, particularly those for household level income and consumption, which had not been used in previous 10/66 DRG surveys.

3. The statistical modelling procedures, and the justification for choice of particular regression models for particular outcomes

1. Catchment area sites

1.1 Geographic locations, and sampling for the 10/66 DRG baseline and follow-up surveys

The INDEP quantitative study sampled households from previous baseline and incidence waves of the 10/66 catchment area surveys in Peru, Mexico, and China. For more details on the 10/66 surveys, please see our recently published cohort profile (Prina et al., 2016). Urban sites in Peru were Lima Cercado and San Miguel in the capital city, Lima (1381 older people sampled for the baseline survey, conducted in 2005; incidence wave, conducted in 2008, with n=890 reinterviewed); rural sites were Cerro Azul, Imperial, Nuevo Imperial, Quilmana, San Luis, and San Vicente in Canete coastal province (baseline survey, 2006, n=552; incidence wave, 2009, n=421). The urban sites in Mexico comprised six districts in Tlalpan, Mexico City (baseline survey, 2006, n=1003 in; incidence wave, 2009, n=749); the rural sites comprised nine villages in Morelos, a mountainous district 70km from Mexico City (baseline survey, 2006, n=1000; incidence wave, 2009, n=713). The urban site in China was Xicheng, close to Tiananmen Square in Beijing City (baseline survey, 2004, n=1160; incidence wave, 2009, n=741); the rural site comprised 14 villages in Daxing, a rural district 40 kilometres away (baseline survey, 2004, n=1002; incidence wave, 2009, n=711).

1.2 Changes in economic development, local infrastructure, and social protection, since the 10/66 baseline survey

Over the ten years since the sites were originally selected (2003-2006) several have undergone significant change and development. This is most evident for the rural China site, where in the context of national economic reforms, land ownership has been granted, and agricultural land has been sold off for property development and infrastructure projects. Beijing’s second airport is soon to be constructed in the district. The urban China site, in the heart of Beijing city, has been affected by construction projects linked to the Beijing Olympics, accounting for the difficulty in tracing participants from previous waves of the 10/66 survey. The rural Peru site was severely affected by an earthquake in August 2007, and reconstruction is still under way. An earlier, descriptive analysis of the economic and social circumstances of older person’s households revealed important differences among the INDEP countries and sites, reflecting the distinctive characteristics of urban and rural communities, and the impact of national policies on the reach of social protection (Prince et al., 2016).

All three countries have benefited from sustained high levels of economic growth over the last decade, around 4-6% per annum in Mexico and Peru, and 8-10% per annum in China. Economic growth has enabled countries across Latin America to pay down national debt while investing more in social protection. China, too, has made great strides in improving pension coverage and reforming healthcare finance (Pozen, 2013). Nevertheless, in all three countries, the fragmented architecture of pension and social assistance programs has resulted in gaps in coverage, inequality and unfairness (Wang, Beland, & Zhang, 2014; Bosch, Melguizo, & Pages, 2013; Rofman & Oliveri, 2012). In Latin America generally, coverage by contributory pension schemes is low because of the ceiling imposed by the dominant informal labour sector; coverage by contributory pensions, around 40%, has been boosted to 60% by the recent rise in non-contributory pension schemes, but these are generally of much lower value (Bosch et al., 2013; Rofman & Oliveri, 2012). In Mexico City, a universal social pension (currently $65 per month) was introduced for all those aged 70 and over in 2001. The federal system of conditional cash transfers (‘Oportunidades’) provides around $22 per month for older people among the poorest families. A federal system of social pensions ’70 y más’ has been rolled out state by state starting with rural communities (population <2500) from 2007, and extended in 2011 to communities with a population of less than 30,000. With the election of the Institutional Revolutionary Party in 2012, this program will be extended to some poor urban neighborhoods and to those aged 65 years and over. The 500 Mexican Pesos ($34) per month provided to the beneficiaries of the 70 y más program can be compared with the average of 17,500 Mexican pesos ($1174) per month for beneficiaries of some contributory schemes. In Peru, as with Mexico, the formal labour sector is smaller than the Latin American average, and pension coverage has historically been among the lowest in Latin America (Rofman & Oliveri, 2012). After the last election the government of President Humala committed to the introduction of means tested pensions of $40 a month (Pension 65) for all those with no contributory pension entitlement. In China there are essentially four types of pension scheme (Pozen, 2013; Wang et al., 2014); 1) before 1997, state-owned enterprises (SOEs) provided non-contributory pensions, now referred to as ‘legacy pensions’ – this then evolved into a contributory Urban Enterprise Pension System (UEPS) covering 280 million urban workers, mainly employees of large private enterprises and State-Owned Enterprises; 2) the Rural Pension social endowment scheme, rolled out from 2009-2012 allows rural workers to make voluntary contributions to individual accounts that are subsidized by local and central government – those aged 60 and over at inception of the scheme could still benefit without contributions, so long as their children chose to contribute; 3) from 2011, a similar old age insurance scheme was available for urban residents not eligible for the UEPS; and 4) a non-contributory pension for civil servants, now being reformed towards a contributory system with 8% salary contributions from employees.

We were able to track changes in pension coverage for older residents, since the baseline 10/66 DRG surveys. The historic urban bias of public policy in China has led to a gross disparity in pension coverage, clearly demonstrated in the 10/66 urban and rural sites (Liu et al., 2009; Prince et al., 2008). The early retirement ages and comparative generosity of the urban cadres’ legacy and civil service pensions account for the high coverage rates and dominance of pension income in the urban China site. Pension coverage in the rural China site has increased from 3.9% (Prince et al., 2008) in 2003 to 14.8% at the time of the INDEP survey, suggesting very limited uptake of the new rural pension scheme in this district. Pension demand may be low because of the increased profits from farming activity and the recent enrichment of some farming families from land sold off for construction and infrastructure projects, including the construction nearby of Beijing’s second airport. However, these benefits have not been evenly distributed as indicated by a highly skewed income distribution, and the high levels of income inequality. While median household income in the rural China site is higher than that in Beijing City, the 25^th^ centile is lower, and consumption levels remain lower than those of the households in the urban site. In Mexico, pension coverage has remained stable at around 70% in the urban site, but has increased from 25% to 91% in the rural site (Prince et al., 2008), reflecting the impact of the targeted benefits provided by the ‘Oportunidades’ and ‘70 y Más’ schemes. Nevertheless, a significant minority of Mexican households continue to live in poverty, with relatively high rates of indebtedness, economic strain, and negatively perceived economic circumstances. The high rates of catastrophic health spending for these Mexican older persons’ households suggest that, for many of them, Seguro Popular has failed to deliver on its promise of financial protection alongside increased access to healthcare (Knaul et al., 2012). In the Peru sites, pension coverage is low, and has not improved since survey baseline (Prince et al., 2008), probably reflecting the delayed implementation of the Pension 65 program.

2. Measures

A full account of the interviews administered in the INDEP study is provided in our open access protocol paper (Mayston et al., 2014). Variables from three sections of the INDEP interview have been used in the analyses for the current publication, covering household composition, and residents’ roles; household economic evaluation; and an updated assessment of the needs for care of older residents. The detailed household interview comprises:

2.1. Household composition and roles – we recorded the age, sex, marital, educational and occupational status of all residents, and their relationship to the oldest index older person.

2.2. Economic evaluation - Household income and consumption were not assessed in previous 10/66 surveys. INDEP study assessments were developed from questionnaires used in community research into social pensions, poverty and wellbeing in South Africa and Brazil (Lloyd-Sherlock, Barrientos, Moller, & Saboia, 2012). We checked with local investigators the relevance and comprehensiveness of questions regarding sources of income and types of expenditure, and adjusted the questions to reflect local systems.

a. A household assets index covering household goods and amenities (telephone or mobile phone, stove, electricity supply, television, radio or stereo, refrigerator, sewing machine, bicycle, computer, and motor vehicles).

b. Monthly household income was estimated by enquiring about 20 different sources of income and allocating each to an individual resident, or to the household if not specifiable. Income sources were clustered into five groups; pensions (government social pensions, employer pension or retirement annuity), paid work (full or part-time regular or occasional work, or income from a business, and any employment benefits), income from assets (savings, investments, property rents, lodgers), government transfers (unemployment benefit, child support grants, disability benefits, public work schemes) and private transfers (money from religious organisations, non-governmental organizations (NGOs) or charities, gifts or regular payments from family or others outside of the household). Total monthly household income was calculated by summing after tax income across all sources and all residents. This monthly amount was then equivalised by dividing by the modified Organisation for Economic Cooperation and Development (OECD) equivalence scale (1.0 for the first adult, 0.5 for all other adults, and 0.3 for children) to account for economies of scale, and converted into 2011 international dollars using PPP exchange rates (The World Bank, 2014).

d. Consumption, 25 items eliciting food consumption (the value or cost of all food consumed at home and outside of the home), household expenses (rent or mortgage, rates and utilities) and other expenditure (clothing, personal items, transport, alcohol, tobacco, business investment, schooling, club membership, hire purchase and debt repayments, holidays and entertainment, lotteries and gambling, private transfers to other households, and charitable donations), also divided by the OECD equivalence scale.

e. Consistent with convention, health and social care expenses were not included in general consumption, but considered separately. These included the self-reported costs of consultations with primary care or hospital doctors, private doctors, traditional healers, inpatient admission and medication costs, and for any paid nursing or social care within the home. Catastrophic healthcare costs were defined as spending more than10% of household income in the last three months on health care.

e. Indicators of household financial strain over the last three years. The ten indicators comprised; asking for help from friends or relatives, an employer, a religious organisation, or charity; taking a loan; cutting down on food consumption; seeking extra work; running up an account with a shop; applying for a grant; apply for food parcels or vouchers; drawing on savings, selling stocks or shares; any other action to address the financial difficulty. The number of indicators endorsed was grouped into three categories for the analyses; none, one and two or more.

f. Subjective assessment of overall financial status; How would you rate the financial situation of this household at present? For the purpose of analysis this was grouped into three categories, very good or good, average, and bad or very bad.

2.3. Needs for care

The main purpose of the INDEP key informant interview was to update the assessment of the older person’s needs for care. The informant is first asked whether the older person requires extra help, support or supervision, because of a health condition or disability, and about critical intervals of care. Seven additional open-ended questions were used to inform a final interviewer rating that the older person does not need care; needs care occasionally; or needs care much of the time (Sousa et al., 2010).

3. Statistical analyses

Regression models were selected depending on the distributional characteristics of the outcome data, based upon tests for over-dispersion, zero-inflation and goodness of fit. Negative binomial regression (generating count ratios) was used for the main income and consumption outcomes, which were generally over-dispersed. Zero-inflated negative binomial regression (generating count ratios) was used for household income from paid work and private transfers, and household healthcare expenditure, which were also characterized by excess zeros. Ordinal regression (odds ratios across ordinal categories) was used for the two ordinal scales (economic strain and dissatisfaction with economic circumstances). Poisson regression (generating prevalence ratios) was used for the two dichotomous outcomes, catastrophic healthcare expenditure and giving up work or education to care for an older person.

Reference List

Bosch, M., Melguizo, A., & Pages, C. (2013). *Better Pensions Better Jobs. Towards universal coverage in Latin America and the Caribbean* Inter-American Development Bank.

Knaul, F. M., Gonzalez-Pier, E., Gomez-Dantes, O., Garcia-Junco, D., Arreola-Ornelas, H., Barraza-Llorens, M. et al. (2012). The quest for universal health coverage: achieving social protection for all in Mexico. *Lancet., 380,* 1259-1279.

Liu, Z., Albanese, E., Li, S., Huang, Y., Ferri, C. P., Yan, F. et al. (2009). Chronic disease prevalence and care among the elderly in urban and rural Beijing, China - a 10/66 Dementia Research Group cross-sectional survey. *BMC.Public Health, 9,* 394.

Lloyd-Sherlock, P., Barrientos, A., Moller, V., & Saboia, J. (2012). Pensions, poverty and wellbeing in later life: Comparative research from South Africa and Brazil. *Journal of Aging Studies, 26,* 243-252.

Mayston, R., Guerra, M., Huang, Y., Sosa, A. L., Uwakwe, R., Acosta, I. et al. (2014). Exploring the economic and social effects of care dependence in later life: protocol for the 10/66 research group INDEP study. *Springerplus., 3:379. doi: 10.1186/2193-1801-3-379. eCollection;%2014.,* 379-3.

Pozen, R. C. (2013). *Tackling the Chinese Pension System* Chicago: The Paulson Institute.

Prina, A. M., Acosta, D., Acostas, I., Guerra, M., Huang, Y., Jotheeswaran, A. T. et al. (2016). Cohort Profile: The 10/66 study. *Int.J.Epidemiol.,* dyw056.

Prince, M., Acosta, D., Albanese, E., Arizaga, R., Ferri, C. P., Guerra, M. et al. (2008). Ageing and dementia in low and middle income countries-Using research to engage with public and policy makers. *Int.Rev.Psychiatry, 20,* 332-343.

Prince, M. J., Lloyd-sherlock, P., Guerra, M., Huang, Y., Sosa, A. L., Uwakwe, R. et al. (2016). The economic status of older peoples households in urban and rural settings in Peru, Mexico and China: a 10/66 INDEP study cross-sectional survey. *SpringerPlus, 5,* 1-16.

Rofman, R. & Oliveri, M. L. (2012). *Pension Coverage in Latin America* (Rep. No. 1217). Washington DC: World Bank.

Sousa, R. M., Ferri, C. P., Acosta, D., Guerra, M., Huang, Y., Ks, J. et al. (2010). The contribution of chronic diseases to the prevalence of dependence among older people in Latin America, China and India: a 10/66 Dementia Research Group population-based survey. *BMC.Geriatr., 10,* 53.

The World Bank (2014). *Purchasing Power Parities and Real Expenditures of World Economies. Summary of Results and Findings of the 2011 International Comparison Program* Washington DC: International Bank for Reconstruction and Development/ The World Bank.

Wang, L., Beland, D., & Zhang, S. (2014). Pension fairness in China. *China Economic Review, 28,* 25-36.
